# Synthesis of an *N*‐Galactosyl Norbornane Aziridine and its Potent Mixed Inhibition of *Aspergillus oryzae* β‐Galactosidase

**DOI:** 10.1002/cbic.202500623

**Published:** 2025-10-27

**Authors:** Aaron McCormack, Ronan Gavin, Mikael Bols, Paul V. Murphy

**Affiliations:** ^1^ School of Biological and Chemical Sciences University of Galway University Road Galway H91TK33 Ireland; ^2^ Department of Chemistry University of Copenhagen Universitetsparken 5 DK‐2100 Copenhagen Denmark; ^3^ SSPC Research Ireland Centre for Pharmaceuticals University of Galway University Road Galway H91TK33 Ireland

**Keywords:** azide alkene cycloaddition, aziridine, diastereoselective, glycosidase, mixed inhibition

## Abstract

Aziridine‐bearing cyclic polyols are established as irreversible covalent inhibitors of glycosyl hydrolases and have been employed as activity‐based probes. In the present study, the stereoselective synthesis of a novel *N*‐galactosyl aziridine derivative on a norbornane scaffold and its subsequent evaluation as an inhibitor of glycosyl hydrolase activity are described. The diastereoselective Huisgen cycloaddition between 2,3,4,6‐tetra‐*O*‐acetyl‐β‐d‐galactopyranosyl azide and norbornene yields *exo*‐triazoline intermediates of norbornane. Following the removal of the acetyl protecting groups and a silica gel‐mediated decomposition of the triazoline intermediates, *exo* *N*‐galactopyranosyl norbornane aziridine (*N*‐(β‐d‐galactopyranosyl)‐*exo*‐3‐azatricyclo[3.2.1.0^2,4^]octane, NGNA) is obtained. Enzymatic assays demonstrate that NGNA exerts potent mixed‐mode inhibition of *Aspergillus oryzae* β‐galactosidase, while exhibiting significant selectivity over green coffee bean α‐galactosidase.

## Graphical abstract

An *N*‐galactosyl aziridine is found to have μM range mixed inhibition constants (*K*_i_ and *K*_i_’) for a fungal β‐galactosidase.

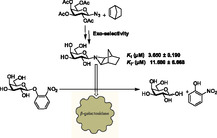

## 1 Introduction

Glycosyl hydrolases, also known as glycosidases, are carbohydrate‐processing enzymes that catalyze the hydrolysis of glycosidic bonds. Glycosidases play an important role in biological processes and have been the subject of much research.^[^ ^1^ ^]^ Glycosidase mechanism involves general acid catalysis and results in either retention or inversion of anomeric configuration.^[^ ^2^ ^]^ A wide range of carbohydrate configurations are found in nature, and glycosidases have evolved great functional diversity.^[^ ^3^ ^]^ Galactosidases, for example, have high specificity for hydrolysis of galactosides and often do not tolerate modifications in the ring, other than at the anomeric carbon.^[^ ^4^ ^]^ *E. coli* β‐galactosidase cleaves lactose into galactose and glucose, and the transglycosylation of lactose into allolactose.^[^ ^4^ ^]^ *A. oryzae* β‐galactosidase is one of the GH35 family of glycosyl hydrolases, meaning it is a retaining galactosidase and exhibits a Koshland double displacement mechanism when hydrolyzing its substrate.^[^ ^2^ ^,^ ^5^, ^6^ ^–^ ^9^ ^]^

**Figure 1:**
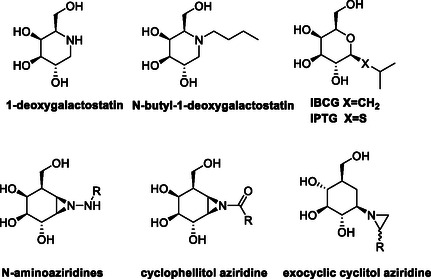
Selected examples of glycosidase inhibitors.

Inhibitors of β‐galactosidase have applications as therapeutics, such as being chaperones for therapy of lysosomal storage disorders^[^ ^7^ ^,^ ^8^ ^]^ and as activity‐based probes.^[^ ^9^ ^]^ Relevant lysosomal storage disorders include GM1‐gangliosidoses and Morquio B syndrome.^[^ ^10^ ^,^ ^11^ ^]^ Lysosomal storage disorders are caused by a mutation of the *GLB1* gene which codes for β‐galactosidase in humans, leading to a deficiency of functioning lysosomal enzymes. Inhibitors of β‐galactosidase can act as substrate reduction therapies^[^ ^12^ ^]^ and pharmacological chaperones, which are often combined with enzyme replacement therapies.^[^ ^10^ ^]^ Galactosidase inhibitors acting as chaperones are believed to bind to mutant enzymes and aid restoration of the active site's catalytic conformation; the promotion of folding of the enzyme to its active conformation can lead to reduced degradation of the enzyme and an increase in its activity within cells.^[^ ^8^ ^,^ ^13^ ^]^ Iminosugars such as 1‐deoxygalactonojirimycin (1‐deoxygalactostatin^[^ ^14^ ^]^) and *N*‐butyl 1‐1‐deoxygalactostatin (**Figure** **1**), which are transition state mimics, have been found to restore mutant β‐galactosidase enzyme activity in vivo^[^ ^15^ ^]^ (Figure 1). Substrate analogs such as isopropyl β‐d‐1‐thiogalactopyranoside (IPTG) and its C‐glycoside analog (IBCG) are also inhibitors of β‐galactosidase;^[^ ^16^ ^,^ ^17^ ^]^ substrate analogs are typically less potent than transition state analogs.^[^ ^18^ ^]^

**Scheme 1:**
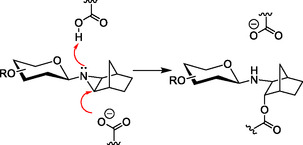
Possible glycosidase inactivation by an N‐glycosyl aziridine

Aziridine derivatives of polyhydroxylated compounds have received attention as covalent inhibitors and activity‐based probes of glycosidases,^[^ ^19^ ^–^ ^21^ ^]^ while reversible aziridine inhibitors have not been frequently reported. More attention has been given to epoxide‐based inhibitors than aziridines, but the latter are known.^[^ ^22^ ^]^ Covalent inhibition arises from the formation of covalent bonds with appropriate amino acid residues in the enzyme's active site, leading to deactivation.^[^ ^23^ ^]^ This type of inhibition can be both reversible and irreversible.^[^ ^24^ ^]^ **Scheme** **1** shows a potential reaction of an *N*‐glycosyl aziridine with the glycosidase active site. Aziridine‐based covalent glycosidase inhibitors hold a potential advantage over epoxide analogs, if they displayed higher initial affinity for the active site, which could arise due to enhanced noncovalent interactions with the negatively charged carboxylate residu‐e in the active site after protonation of the aziridine N atom.^[^ ^22^ ^]^ Alcaide et al. report the synthesis and evaluation of galacto‐configured *N*‐aminoaziridines (Figure 1), which were found to be potent inhibitors of β‐galactosidase from *E. coli* and *A. oryzae*.^[^ ^25^ ^]^ Furthermore, β‐d‐galactopyranose‐configured derivatives of cyclophellitol aziridine have been designed and synthesized as putative structure–activity probes for β‐galactosidase (Figure 1).^[^ ^20^ ^]^ While *exo*‐cyclic aziridines have been synthesized as potential β‐glucosidase inhibitors (Figure 1),^[^ ^26^ ^]^ their inhibitory properties have not yet been reported.

Here, we show the synthesis of an *N*‐galactopyranosyl aziridine via a diastereoselective glycosyl azide‐norbornene cycloaddition (Huisgen cycloaddition reaction) and subsequent decomposition of triazoline intermediates. The new aziridine showed potent μM range inhibition of the β‐d‐galactosidase from *A. oryzae*.

## 2 Results and Discussion

### 2.1 Diastereoselective Synthesis of N‐Galactosyl Aziridine 6

**Scheme 2:**
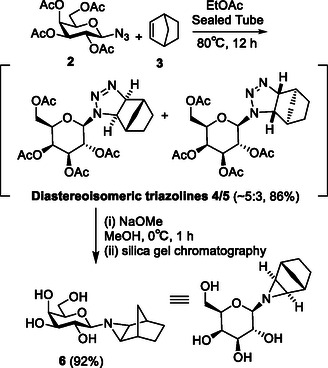
Synthesis of *exo* aziridine **6.**

The synthesis, shown in **Scheme** **2**, began from acetylated β‐d‐galactopyranosyl azide **2**,^[^ ^27^ ^]^ which was reacted with norbornene **3** in a sealed glass pressure tube in EtOAc at 80 °C for 12 h and gave two *exo*‐diastereoisomers **4** (second eluted product) and **5** (first eluted product) (≈5:3 ratio, 86% isolated yield) after chromatography of the reaction residue. *Endo*‐diastereoisomers were not detected in the reaction mixture, and the two *exo*‐isomers were separable. Deacetylation of **4** and **5** was performed using NaOMe in MeOH at 0 °C (Zemplén procedure); ^13^C‐NMR analysis of the reaction mixtures before chromatography indicated that two fully deacetylated *N*‐galactopyranosyl triazolines were initially obtained by the Zemplén procedure; however, silica gel chromatography of the triazoline mixture led to their decomposition to afford a single *exo*‐aziridine **6** in 92% yield. The decomposition of triazolines to aziridines is known,^[^ ^28^ ^,^ ^29^ ^]^ and the acidity of silica gel catalyzes this transformation. This reaction likely proceeds via protonation of the anomeric nitrogen, then N‐N bond cleavage to give a diazonium ion, followed by norbornyl cation formation with N_2_ loss, and subsequent formation of **6**; this mechanism would account for the observed retention of configuration.

NMR spectral data and high‐resolution mass spectrometry (HRMS) was used to assign structures to triazolines and aziridine **6**; the NMR data obtained showed consistency with that reported for norbornene‐derived triazolines described earlier in the literature.^[^ ^30^, ^31^ ^–^ ^32^ ^]^ The ^13^C‐NMR chemical shift of the CH groups (*δ* 89.85, 87.37 ppm) directly adjacent to the triazoline N=N group provided support to assignment of triazoline structures to **4/5**, rather than aziridines, and the experimentally determined chemical shifts also showed consistency with density functional theory (DFT)‐calculated chemical shifts (see Supporting Information). Also, HRMS analysis of **4**/**5** supported the assignment of triazolines to these substances. Initial liquid chromatography (LC)‐HRMS investigations found that both **4** and **5** were decomposed in formic acid buffer (used to enhance ionization of compounds) to an aziridine. However, direct injections of the solutions avoiding acidic buffer led to the detection of the required molecular masses for the triazoline adducts, which were within 5 ppm accuracy of the calculated mass.

Exclusive formation of the *exo*‐diastereoisomer from 1,3‐dipolar cycloadditions with 2‐norbornene has been established initially by Huisgen et al.,^[^ ^32^ ^]^ and *exo*‐products have been reported by others subsequently.^[^ ^30^ ^,^ ^33^ ^]^ Huisgen suggested that steric hindrance is greater when the azide approaches from the *endo* face, contributing to the formation of the *exo*‐isomer. Later, Lopez and Houk used DFT to generate *exo‐* and *endo‐*transition states^[^ ^34^ ^]^ and found that selectivity for the *exo*‐diastereoisomer over *endo* from norbornene is influenced by a reduction of torsional strain in the *exo*‐transition state compared to that in the *endo*‐TS isomer. To our knowledge, there have not been examples in the literature indicating that *endo*‐isomer formation is competitive with that of *exo* from reactions involving azides, although a mixture of *exo‐* and *endo‐*products was formed for the reaction of nitrile oxides with norbornadiene.^[^ ^35^ ^]^ DFT calculations using Gaussian^[^ ^36^ ^]^ were used to predict ^13^C NMR chemical shifts for *exo‐* and *endo‐*aziridine diastereoisomers derived from 2‐norbornene; a major difference was predicted for the ^13^C chemical shift for the bridging methylene carbon atom between the *exo‐* (calculated = *δ* 31.0 ppm) and *endo*‐diastereoisomer (calculated = *δ* 53.7 ppm). We thus assigned the structure to the *exo*‐isomer for **6** based on the product having its ^13^C chemical shift for the bridging methylene at 29.3 ppm, much closer in magnitude to that predicted for the *exo*‐isomer by the DFT method (see Supporting Information for more details).

### 2.2 Evaluation of β‐Galactosidase Inhibition

With *N*‐galactosyl aziridine **6** in hand, galactosidase inhibition assays were carried out which followed a similar method described by Bols et al.^[^ ^37^ ^]^ We first tested **6** for inhibition of the hydrolysis of 2‐nitrophenyl‐β‐d‐galactopyranoside by β‐galactosidase from *A. oryzae*. The assay was carried out at 37 °C in pH 6 phosphate buffer, and absorbance was measured at 400 nm to monitor the formation of 2‐nitro‐phenol over 10 min. The assay was initially performed without the presence of inhibitor. A steady increase in the amount of product forming was observed at all substrate concentrations. Measurements were then carried out using different concentrations of inhibitor [I]. Plots of absorbance versus time when [I] = 2, 1, 0.1, and 0.01 mM clearly indicated a reduction in the enzyme's activity (see Supporting Information).

Lineweaver–Burk plots (1/V vs. 1/[S]) were constructed for the four different concentrations of **6** used and for the enzymatic reaction without inhibitor (see Supporting Information). Analysis of the plots showed that compound **6** was not acting as a conventional competitive inhibitor, nor as a pure noncompetitive inhibitor. Given that noncompetitive inhibition occurs when *K*_i _= *K*_i_′, while mixed inhibition—a subcase of noncompetitive inhibition—occurs when *K*_i _≠ *K*_i_′, aziridine **6** was determined to be acting as a mixed inhibitor of β‐galactosidase. Furthermore, a Cornish‐Bowden plot (direct linear plot) was in good agreement with the expected plot of a mixed inhibitor (see Supporting Information).^[^ ^38^ ^]^ In a recent paper, Pesaresi argued that mixed inhibition may often be misidentified as competitive inhibition. Pesaresi showed that apparent mixed inhibition could be observed for inhibitors not binding to an allosteric site,^[^ ^39^ ^]^ in contrast to definition commonly used in textbooks.^[^ ^40^ ^]^ Five different cases/mechanisms whereby analysis via Michaelis–Menten steady‐state kinetics can give the impression of mixed inhibition were suggested. The cases proposed were as follows: I) the inhibitor binding was sufficiently strong that it required testing at a lower concentration than the enzyme, II) time‐dependent inhibition, III) the enzyme‐catalyzed reaction was a multisubstrate reaction, IV) regeneration of the enzyme was a slow process, or V) the inhibitor bound to an *exo* site (i.e., a substrate‐binding site outside the active site).

Based on the Lineweaver–Burk plot of the lowest [I] (0.01 mM), *K*_i_ and *K*_i_′ values for the mixed inhibition exhibited by **6** were calculated. *V*_max_ (y‐intercept) is considered to remain unchanged for *K*_i_, while *K*_m_′ (slope) changes in response to an inhibitor. Therefore, (Formula 1) was used, giving *K*_i_ = 3.65 µM (**Table** **1**). Analogously for *K*_i_′, *K*_m_′ is considered to remain unchanged, while *V*_max_ changes in response to an inhibitor. Formula (2) was used and gave *K*_i_′ = 11.68 µM (Table 1).

**Table 1:** *K*_i_ and *K*_i_′ values for compound 6 in mM at 37 °C.

	Enzyme	*K*_i_ [µM]	*K*_i_′ [µM]
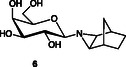	β‐Galactosidase (*A. oryzae*)	3.650(±0.190)	11.680 (±6.668)
	β‐Galactosidase (green coffee beans)	2247 (±240)	3510 (±702)

(1) α=1+[I]/Ki(α=slopeinhibitorslopenoinhibitor)

(2) α′=1+[I]/Ki′(α′=y−interceptinhibitory‐interceptnoinhibitor)

The crystal structure of β‐galactosidase from *Aspergillus ozyzae* has been previously elucidated at 2.60 Å by Maksimainen et al.^[^ ^5^ ^]^ However, there was no evidence that it possessed an allosteric or *exo*‐binding site.^5^ While this finding did not mean allosteric binding could be excluded, Pesaresi's explanations for mixed inhibition were of interest and considered. Case I could readily be dismissed because testing in our assay was conducted at [I] >> [E]. Case II could also be dismissed because time‐dependent inhibition was not observed as was evident from the linear progress curves (see Supporting Information). Case III assumed a multisubstrate reaction which was not relevant here. Case IV assumed a mechanism where recovery of the enzyme from product release is slow. It was possible in this case that reprotonation of the enzyme could be slow, however. Case V assumed binding to an *exo* site which is not very different from allosteric binding. We suspected that Pesaresi's cases IV and V were the most likely reasons for the observed kinetics.

### 2.3 β‐Galactosidase Inactivation Assay

While the assay results showed that **6** acted as a mixed inhibitor of β‐galactosidase, it was clear that the inhibitor could still be binding to the active site. Therefore, an investigation was carried out to determine whether the inhibitor could also be deactivating the enzyme via covalent inhibition, i.e., inactivating the enzyme's catalytic site. An assay was designed to determine whether the activity of the enzyme decreased over time while incubated with inhibitor **6**. A decrease in the β‐galactosidase's activity would indicate that the inhibitor was binding covalently and causing inactivation. A similar procedure described by Caron and Withers was used for this assay.^[^ ^19^ ^]^ The inhibitor was incubated at 37 °C with the enzyme, and aliquots of the mixture were added to a plate containing a single substrate concentration (12 mM) over 1 h. The absorbance was then measured as before. Two different concentrations of inhibitors (1 and 0.01 mM) were incubated with the enzyme, while water was used as a control to compare. The enzymatic reaction rate (*V*) after adding the enzyme–inhibitor mixture was once again determined by measuring the amount if 2‐nitro‐phenol produced over time (10 min) at 37 °C in phosphate buffer (pH 6). *V* was measured at three different time points for each sample: T0_min_, T30_min_, and T60_min_. As shown by Caron and Withers, a plot of ln(*V*) versus time was constructed to determine whether the enzyme activity had been affected after incubation with the inhibitor. When [I] = 0.01 mM, there did not appear to be any measurable decrease in enzyme activity over time compared to the control sample as the ln(*V*) showed no trend of decreasing. When [I] = 1 mM, there appeared to be a slight decrease in enzyme activity over time, indicated by a gradual downward slope. However, the irreversible inhibition component is very small and insignificant.

### 2.4 β‐Galactosidase Inhibition Assay

To determine whether *N*‐galactosyl aziridine **6** selectively inhibited the β‐galactosidase enzyme, an inhibition assay was also performed with β‐galactosidase from green coffee beans. β‐Galactosidases have selectivity for galactosides with an β‐configuration at the anomeric position.^[^ ^41^ ^]^ This enzyme is also a retaining glycosidase, operating via the same mechanism as the β‐galactosidase used previously.^[^ ^42^ ^]^ The inhibition constants for mixed inhibition *K*_i_ and *K*_i_′ were calculated as before from the Lineweaver–Burk plot (see Supporting Information). As shown in Table 1, it was found that *K*_i_ = 2247 μM, while *K*_i_′ = 3510 μM when [I] = 1 mM. These results indicated that **6** was not a potent mixed inhibitor of β‐galactosidase and showed high selectivity for inhibition of β‐galactosidase.

## 3 Conclusion

We have described synthetic details to an *N*‐galactosyl aziridine derivative of norbornene that acts as a mixed inhibitor of β‐galactosidase. The facile synthesis involved diastereoselective azide–alkene cycloaddition, performed in a sealed tube, from readily obtained 2,3,4,6‐tetra‐*O*‐acetyl‐*β*‐d‐galactopyranosyl azide with norbornene which gave *exo*‐diastereoisomeric triazolines. Subsequent protecting group removal from the triazolines and treatment with silica gel gave the aziridine. Further improvements to the synthesis work can be envisaged, such as use of continuous flow chemistry^[^ ^43^ ^]^ which we have explored to increase the efficiency and safety of the azide–alkene cycloaddition.^[^ ^44^ ^,^ ^45^ ^]^ The testing of **6** as an inhibitor of β‐galactosidase (*A. oryzae*) showed it to be a potent (μM range) mixed inhibitor. An inhibition assay with β‐galactosidase from green coffee beans showed **6** was a weak inhibitor and exhibited high selectivity as an inhibitor of β‐galactosidase. An enzyme–inhibitor incubation assay did not show that **6** was acting as a covalent inhibitor. Future structure–activity relationship studies could elucidate the determinants of potency and selectivity, while assessment of NGNA against a wider range of retaining galactosidases may reveal its potential as a probe for glycoscience research.

## 4 Experimental Section

4.1

4.1.1

##### Mixture of (*2R,3S,4S,5R,6R*)‐2‐(acetoxymethyl)‐6‐((*3aS,4R,7S,7aR*)‐3a,4,5,6,7,7a‐hexahydro‐1H‐4,7‐methanobenzo[d[1–3] triazol‐1‐yl)tetrahydro‐2H‐pyran‐3,4,5‐triyl triacetate and (*2R,3S,4S,5R,6R*)‐2‐(acetoxymethyl)‐6‐((*3aS,4R,7S,7aR*)‐3a,4,5,6,7,7a‐hexahydro‐1H‐4,7‐methanobenzo[d[1–3] triazol‐1‐yl)tetrahydro‐2H‐pyran‐3,4,5‐triyl triacetate (4/5)

**Figure 2:**
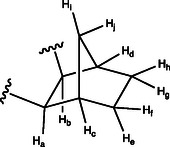
Labeling of norbornane moiety protons used in NMR spectroscopic signal assignments.

Compound **2** (1.50 g, 4.02 mmol) was added to a 15 mL Ace pressure tube (heavy‐wall borosilicate glass tubes, Sigma–Aldrich) and dissolved in EtOAc (3 mL). To the solution was added 2‐norbornene **3** (0.57 g, 6.03 mmol) and the sealed tube was stirred vigorously at 80 °C. ^1^H‐NMR spectroscopic analysis indicated that the reaction was completed after 12 h. *Caution: Ace pressure tubes are not guaranteed against breakage caused by pressure or vacuum and should not be used if scratched or otherwise damaged. It is not advisable to exceed the quantities of azide* ***2*** *given here when using Ace pressure tubes (rating of 150 psig) given triazolines formed in the reaction have potential to react further to liberate nitrogen gas.* The tubes were cooled before opening, which was done carefully and in a fume cupboard. The mixture was concentrated in vacuo and silica gel chromatography gave the title compounds **4**/**5** (≈5:3, 1.55 g, 86%—combined yield) were both afforded as white solids (**Figure** **2**). The chromatography was performed using gradient elution (cyclohexane‐EtOAc 5:1 to 4:1), and the silica gel had been preneutralized using 3% Et_3_N.

**Analytical data for major isomer 4 (eluted as the second isomer):** R_*f*_ 0.33 (cyclohexane‐EtOAc 3:2); ^1^H‐NMR (400 MHz, CDCl_3_) *δ:* 5.57 (apt t, 1H, ^3^J_2,1_ 9.3 Hz, ^3^J_2,3_ 9.9 Hz, H‐2), 5.44 (dd, 1H, ^3^J_4,5_ 1.4 Hz, ^3^J_4,3_ 3.2 Hz, H‐4), 5.29 (d, 1H, ^3^J_1,2_ 9.3 Hz, H‐1), 5.13 (d, 1H, ^3^J_3,4_ 3.2 Hz, ^3^J_3,2_ 9.9 Hz, H‐3), 4.44 (d, 1H, ^3^J_b,a_ 9.3 Hz, H_b_), 4.10 (overlapping signals, 2H, H‐6a and H‐6b), 3.99 (apt td, 1H, ^3^J_5,4_ 1.4 Hz, ^3^J_5,6a_ 6.6 Hz, ^3^J_5,6b_ 7.0 Hz, H‐5), 3.47 (d, 1H, ^3^J_a,b_ 9.3 Hz, H_a_), 2.65 (d, 1H, ^3^J_d,g_ 4.1 Hz, H_d_), 2.47 (d, 1H, ^3^J_c, f_ 4.1 Hz, H_c_), 2.17 (s, 3H, OAc), 2.04 (s, 3H, OAc), 2.01 (s, 3H, OAc), 2.00 (s, 3H, OAc), 1.54 (m, 1H, H_g_), 1.52 (m, 1H, H_f_), 1.28 (m, 1H, H_h_), 1.20 (m, 1H, H_e_), 1.14 (d, 1H, ^2^J_i,j_ 10.8 Hz, H_i_), 1.09 (d, 1H, ^3^J_j,i_ 10.8 Hz, H_j_); ^13^C‐NMR (101 MHz, CDCl_3_) *δ*: 170.55 (**C=**O, OAc), 170.31 (**C=**O, OAc), 170.19 (**C=**O, OAc), 169.75 (**C=**O, OAc), 87.37 (C_b_), 86.41 (C‐1), 72.14 (C‐5), 71.99 (C‐3), 67.26 (C‐4), 66.29 (C‐2), 61.34 (C‐6), 59.14 (C_a_), 42.34 (C_c_), 40.95 (C_d_), 33.38 (**C**H_i_H_j_), 25.97 (**C**H_g_H_h_), 25.01 (**C**H_e_H_f_), 20.85 (**C**H_3_, OAc), 20.85 (**C**H_3_, OAc), 20.81 (**C**H_3_, OAc), 20.71 (**C**H_3_, OAc); FTIR cm^−1^: 2964, 1743, 1368, 1212, 1043, 979, 952, 936, 891, 845, 765; HRMS (ESI) *m/z* calc for C_21_H_29_NO_9_Na: 462.1734, found 462.1742 [M + Na]^+^.

**Analytical data for minor isomer 5 (first eluted compound):** R_*f*_ 0.35 (cyclohexane:EtOAc 3:2); ^**1**^H‐NMR (400 MHz, CDCl_3_) *δ:* 5.42 (m, 1H, H‐4), 5.40 (m, 1H, H‐1), 5.33 (apt t, 1H, ^3^J_2,1_ 9.3 Hz, ^3^J_2,3_ 9.6 Hz, H‐2), 5.10 (dd, 1H, ^3^J_3,4_ 3.4 Hz, ^3^J_3,2_ 9.6 Hz, H‐3), 4.54 (d, 1H, ^3^J_b,a_ 9.1 Hz, H_b_), 4.08 (overlapping signals, 2H, H‐6a and H‐6b), 4.01 (apt td, 1H, ^3^J_5,4_ 1.1 Hz, ^3^J_5,6a _= ^3^J_5,6b_ 6.7 Hz, H‐5), 3.46 (d, 1H, ^3^J_a,b_ 9.1 Hz, H_a_), 2.72 (d, 1H, ^3^J_d,g_ 3.7 Hz, H_d_), 2.30 (d, 1H, ^3^J_c,f_ 3.7 Hz, H_c_), 2.18 (s, 3H, OAc), 2.04 (s, 3H, OAc), 2.03 (s, 3H, OAc), 2.00 (s, 3H, OAc), 1.53 (overlapping signals, 2H, H_f_ and H_g_), 1.28 (m, 1H, H_h_), 1.18 (m, 1H, H_e_), 1.11 (d, 1H, ^2^J_ij_ 10.5 Hz, H_i_), 0.99 (d, 1H, ^2^J_j,i_ 10.5 Hz, H_j_); ^13^C‐NMR (101 MHz, CDCl_3_) *δ*: 170.54 (**C**=O, OAc), 170.33 (**C=**O, OAc), 170.22 (**C=**O, OAc), 169.62 (**C=**O, OAc), 89.85 (C_b_), 86.35 (C‐1), 72.55 (C‐3), 72.22 (C‐4), 67.52 (C‐2), 67.32 (C‐5), 61.30 (C‐6), 57.21 (C_a_), 41.81 (C_c_), 40.94 (C_d_), 32.34 (**C**H_i_H_j_), 26.20 (**C**H_g_H_h_), 24.91 (**C**H_e_H_f_), 20.90 (**C**H_3_, OAc), 20.90 (**C**H_3_, OAc), 20.80 (**C**H_3_, OAc), 20.72 (**C**H_3_, OAc); FTIR cm^−1^: 2962, 1738, 1366, 1218, 1036, 1016, 951, 921, 892, 849, 767, 739; HRMS (ESI) *m/z* calc for C_21_H_29_N_3_O_9_Na: 490.1796, found 490.1794 [M + Na]^+^.

##### N‐(β‐d‐Galactopyranosyl)‐Exo‐3‐Azatricyclo[3.2.1.02,4]octane (6)

To acetylated *N*‐galactosyl triazoline mixture **4/5** (0.11 g, 0.24 mmol) in MeOH (5 mL) at 0 °C was added NaOMe (1 equiv). The reaction mixture was stirred for 1 h; thin layer chromatography analysis indicated the reaction reached completion. Subsequent silica gel chromatography of both compounds gave aziridine **6** (0.06 g, 90%). Compound **6** was also formed by treating the solution of deacetylated major or minor triazoline isomer (0.05 g, 0.17 mmol) in MeOH (5 mL) with silica gel (0.20 g) for 2 h. The reaction mixture was concentrated in vacuo and purified using silica gel chromatography (EtOAc‐MeOH 5:1) affording an analytical sample of **6** (0.04 g, 92%) as a white solid.

**Analytical data for deacetylated triazoline major isomer:** ^**1**^H‐NMR (600 MHz, CDCl_3_) *δ:* 4.82 (d, 1H, ^3^J_1,2_ 9.2 Hz, H‐1), 4.38 (d, 1H, ^3^J_b,a_ 9.6 Hz, H_b_), 4.12 (apt t, 1H, ^3^J_2,1_ 9.2 Hz, ^3^J_2,3_ 9.3 Hz, H‐2), 3.91 (dd, 1H, ^3^J_4,5_ 1.1 Hz, ^3^J_4,3_ 3.4 Hz, H‐4), 3.71 (overlapping signals, 2H, H‐6a and H‐6b), 3.65 (d, 1H, ^3^J_a,b_ 9.6 Hz, H_a_), 3.58 (overlapping signals, 1H, H‐5), 3.58 (overlapping signals, 1H, H‐3), 2.55 (d, 1H, ^3^J_d,g_ 4.7 Hz, H_d_), 2.53 (d, 1H, ^3^J_c,f_ 4.4 Hz, H_c_), 1.58 (m, 1H, H_g_), 1.51 (m, 1H, H_f_), 1.34 (m, 1H, H_h_), 1.24 (m, 1H, H_i_), 1.23 (m, 1H, H_e_), 1.17 (m, 1H, H_j_); ^13^C‐NMR (151 MHz, CDCl_3_) *δ*: 89.47 (C‐1), 86.10 (triazoline CH—N=N), 78.23 (C‐5), 75.98 (C‐3), 70.45 (C‐4), 69.02 (C‐2), 62.65 (C‐6), 61.39 (C_a_), 43.23 (C_d_), 42.65 (C_c_), 33.21 (**C**H_i_H_j_), 26.84 (**C**H_g_H_h_), 25.54 (**C**H_e_H_f_); HRMS (ESI) *m/z* calc for C_13_H_21_N_3_O_5_HNa: 322.1373, found 322.1363 [M + Na]^+^.

**Analytical data for deacetylated triazoline minor isomer:** ^**1**^H‐NMR (600 MHz, CDCl_3_) *δ:* 5.06 (d, 1H, ^3^J_1,2_ 9.4 Hz, H‐1), 4.42 (d, 1H, ^3^J_b,a_ 9.5 Hz, H_b_), 3.91 (dd, 1H, ^3^J_4,5_ 1.1 Hz, ^3^J_4,3_ 3.4 Hz, H‐4), 3.85 (t, 1H, ^3^J_2,1 _= ^3^J_2,3_ 9.4 Hz, H‐2), 3.70–3.68 (overlapping signals, 3H, H_a_, H‐6a and H‐6b), 3.62 (td, 1H, ^3^J_5,4_ 1.1 Hz, ^3^J_5,6b_ = ^3^J_5,6a_ = 6.0 Hz, H‐5), 3.57 (dd, 1H, ^3^J_3,4_ 3.4 Hz, ^3^J_3,2_ 9.4 Hz, H‐3), 2.58 (d, 1H, ^3^J_d,g_ 4.3 Hz, H_d_), 2.50 (d, 1H, ^3^J_c,d_ 4.3 Hz, H_c_), 1.57 (m, 1H, H_g_), 1.50 (m, 1H, H_f_), 1.31 (m, 1H, H_h_), 1.24 (m, 1H, H_e_), 1.18 (s, 1H, H_i_), 1.18 (s, 1H, H_j_); ^13^C‐NMR (151 MHz, CDCl_3_) *δ*: 89.65 (C‐1), 88.11 (C_b_), 78.09 (C‐5), 76.25 (C‐3), 70.53 (C‐4), 70.24 (C‐2), 62.41 (C‐6), 59.91 (C_a_), 43.55 (C_c_), 42.39 (C_d_), 33.38 (**C**H_i_H_j_), 26.97 (**C**H_g_H_h_), 25.38 (**C**H_e_H_f_); HRMS (ESI) *m/z* calc for C_13_H_21_N_3_O_5_HNa: 322.1373, found 322.1372 [M + Na]^+^.

**Analytical data for aziridine 6:** R_*f*_ 0.54 (EtOAc‐MeOH 3:1); ^**1**^H‐NMR (400 MHz, CDCl_3_) *δ:* 3.80 (dd, 1H, ^3^J_4,5_ 1.3 Hz, ^3^J_4,3_ 3.3 Hz, H‐4), 3.72 (dd, 1H, ^3^J_6b,5_ 6.6 Hz, ^2^J_6b, 6a_ 11.4 Hz, H‐6b), 3.66 (dd, 1H, ^3^J_6a,5_ 5.2 Hz, ^2^J_6a,6b_ 11.4 Hz, H‐6a), 3.57 (dd, 1H, ^3^J_2,1_ 8.3 Hz, ^3^J_2,3_ 9.7 Hz, H‐2), 3.39 (ms, 2H, H‐3 and H‐5), 3.07 (d, 1H, ^3^J_1,2_ 8.3 Hz, H‐1), 2.35 (s, 1H, H_d_), 2.31 (s, 1H, H_c_), 2.23 (d, 1H, ^3^J_b,a_ 5.7 Hz, H_b_), 2.05 (d, 1H, ^3^J_a,b_ 5.7 Hz, H_a_), 1.50 (d, 1H, ^2^J_i,j_ 9.5 Hz, H_i_), 1.43 (overlapping signals, 2H, H_f_ and H_g_), 1.22 (overlapping signals, 2H, H_e_ and H_h_), 0.66 (d, 1H, ^2^J_j,i_ 9.5 Hz, H_j_); ^13^C‐NMR (101 MHz, CDCl_3_) *δ*: 95.51 (C‐1), 78.34 (C‐5), 75.46 (C‐3), 73.45 (C‐2), 70.55 (C‐4), 62.70 (C‐6), 39.15 (C_b_), 37.37 (C_a_), 37.25 (C_d_), 37.05 (C_c_), 29.33 (**C**HiH_j_), 27.66 (**C**H_g_H_h_), 27.47 (**C**H_e_H_f_); FTIR cm^−1^: 3309, 2908, 1591, 1374, 1334, 1045, 991, 919, 852, 803, 765; HRMS (ESI) *m/z* calc for C_13_H_21_NO_5_H: 272.1492, found 272.1499 [M + H]^+^.

##### Measurements of β‐Galactosidase Inhibition

β‐Galactosidase inhibition assays were performed according to a similar procedure described by Bols et al.^[^ ^37^ ^]^ For each inhibitor and for a control (without inhibitor), 8 wells in a 96‐well plate were used. Each well contained 110 µL of 0.1 M pH 6 phosphate buffer (12.3 mL 0.2 M Na_2_HPO_4_ + 87.7 mL 0.2 M NaH_2_PO_4_ + 100 mL distilled water). 10 to 100 µL of a 2‐nitrophenyl β‐d‐galactopyranoside solution at different concentrations (2.5, 25, and 50 mM) was added to each of the wells to create a range of eight substrate concentrations (0.5 to 15 mM). 20 μL of either the inhibitor (12.5 mM stock solution, 1 mM concentration in well) or distilled water (control) was added to each well. The volume in each well was made up to 230 µL using distilled water. Finally, the reaction was initiated by adding 20 µL of a dilute β‐galactosidase solution (1 mg/mL, β‐galactosidase from *A. oryzae*). The formation of 2‐nitrophenol was monitored for 10 min at 37 °C by measurement of the absorbance at 400 nm using an Epoch microplate reader. After plotting absorbance versus time for the eight reactions with an inhibitor and the eight reactions without an inhibitor, initial velocities (*V*) were calculated from the slopes for each reaction. These values were used to construct two Lineweaver–Burk plots (1/V vs. 1/[S]). Two Michaelis–Menten constants were calculated from these plots: *K*_m_ (without inhibitor) and *K*_m_′ (with inhibitor). The *V*_max_ values with and without inhibitor were also determined. Two inhibition constants, *K*_i_ and *K*_i_′, were calculated from these values.

##### Measurements of β‐Galactosidase Inhibition

β‐Galactosidase assays were carried out according to the same procedure as used previously. For each inhibitor and for a control (without inhibitor), 8 wells in a 96‐well plate were used. Each well contained 110 µL of 0.1 M pH 6 phosphate buffer (12.3 mL 0.2 M Na_2_HPO_4_ + 87.7 mL 0.2 M NaH_2_PO_4_ + 100 mL distilled water). 10 to 100 µL of a 4‐nitrophenyl β‐d‐galactopyranoside solution at different concentrations (2.5 and 25 mM) was added to each of the wells to create a range of eight substrate concentrations (0.2 to 10 mM). 20 µL of either the inhibitor (12.5 mM stock solution, 1 mM concentration in well) or distilled water (control) was added to each well. The volume in each well was made up to 230 µL using distilled water. Finally, the reaction was initiated by adding 20 µL of a dilute β‐d‐galactosidase solution (0.1 mg mL^−^^1^, β‐d‐galactosidase from green coffee beans). The formation of 4‐nitrophenol was monitored for 10 min at 37 °C by measurement of the absorbance at 400 nm using an Epoch microplate reader. After plotting absorbance versus time for the eight reactions with an inhibitor and the eight reactions without an inhibitor, initial velocities (*V*) were calculated from the slopes for each reaction. These values were used to construct two Lineweaver–Burk plots (1/V vs. 1/[S]). Two Michaelis–Menten constants were calculated from these plots: *K*_m_ (without inhibitor) and *K*_m_′ (with inhibitor). The *V*_max_ values with and without inhibitor were also determined. Two inhibition constants, *K*_i_ and *K*_i_′, were calculated from these values.^[^ ^46^, ^47^, ^48^, ^49^, ^50^ ^–^ ^51^ ^]^

## Supporting Information

The authors have cited additional references within the Supporting Information.^[48–53]^ The supporting information contains NMR spectra, additional enzyme kinetics data and calculated chemical shifts.

## Conflict of Interest

The authors declare no conflict of interest.

## Supporting information

Supplementary Material

## Data Availability

The data that support the findings of this study are available from the corresponding author upon reasonable request.

## References

[1] M. P. Kötzler , S. M. Hancock , S. G. Withers , in Encyclopedia of Life Sciences, Wiley, Chichester, UK 2014.

[2] D. E. Koshland , Biol. Rev. 1953, 28, 416.

[3] G. Davies , B. Henrissat , Structure 1995, 3, 853. 8535779 10.1016/S0969-2126(01)00220-9

[4] D. H. Juers , B. W. Matthews , R. E. Huber , Protein Sci. 2012, 21, 1792. 23011886 10.1002/pro.2165 PMC3575911

[5] M. M. Maksimainen , A. Lampio , M. Mertanen , O. Turunen , J. Rouvinen , Int. J. Biol. Macromol. 2013, 60, 109. 23688418 10.1016/j.ijbiomac.2013.05.003

[6] S. Zhang , J. D. McCarter , Y. Okamura‐Oho , F. Yaghi , A. Hinek , S. G. Withers , J. W. Callahan , Biochem. J. 1994, 304, 281. 7998946 10.1042/bj3040281 PMC1137483

[7] Y. Suzuki , Brain Dev. 2013, 35, 515. 23290321 10.1016/j.braindev.2012.12.002

[8] E. M. Sánchez‐Fernández , J. M. G.ía Fernández , C. O. Mellet , Chem. Commun. 2016, 52, 5497. 10.1039/c6cc01564f 27043200

[9] C.‐L. Kuo , Q. Su , A. M. C. H. van den Nieuwendijk , T. J. M. Beenakker , W. A. Offen , L. I. Willems , R. G. Boot , A. J. Sarris , A. R. A. Marques , J. D. C. Codée , G. A. van der Marel , B. I. Florea , G. J. Davies , H. S. Overkleeft , J. M. F. G. Aerts , Org. Biomol. Chem. 2023, 21, 7813. 37724332 10.1039/d3ob01261a

[10] A. K. Rha , A. S. Maguire , D. R. Martin , Appl. Clin. Genet. 2021, 14, 209. 33859490 10.2147/TACG.S206076 PMC8044076

[11] A. Caciotti , S. C. Garman , Y. Rivera‐Colón , E. Procopio , S. Catarzi , L. Ferri , C. Guido , P. Martelli , R. Parini , D. Antuzzi , R. Battini , M. Sibilio , A. Simonati , E. Fontana , A. Salviati , G. Akinci , C. Cereda , C. Dionisi‐Vici , F. Deodato , A. d’Amico , A. d’Azzo , E. Bertini , M. Filocamo , M. Scarpa , M. di Rocco , C. J. Tifft , F. Ciani , S. Gasperini , E. Pasquini , R. Guerrini , et al, Biochem. Biophys. Acta 2011, 1812, 782. 21497194 10.1016/j.bbadis.2011.03.018 PMC3210552

[12] T. D. Butters , R. A. Dwek , F. M. Platt , Glycobiology 2005, 15, 43R. 15901676 10.1093/glycob/cwi076

[13] S. Ishii , H. Chang , K. Kawasaki , K. Yasuda , H. Wu , S. Garman , J. Fan , Biochem. J. 2007, 407, 285. 17555407 10.1042/BJ20070479 PMC1948963

[14] S. Aoyagi , S. Fujimaki , N. Yamazaki , C. Kibayashi , J. Org. Chem. 1991, 56, 815.

[15] L. Tominaga , Y. Ogawa , M. Taniguchi , K. Ohno , J. Matsuda , A. Oshima , Y. Suzuki , E. Nanba , Brain Dev. 2001, 23, 284. 11504597 10.1016/s0387-7604(01)00216-9

[16] E. Hever , V. Santhanam , S. Alberi , A. Dhara , M. Bols , H.‐P. Nasheuer , P. V. Murphy , Org. Biomol. Chem. 2024, 22, 7460. 39189157 10.1039/d4ob01286k

[17] K.‐S. Ko , J. Kruse , N. L. Pohl , Org. Lett. 2003, 5, 1781. 12735776 10.1021/ol034444m

[18] T. M. Gloster , G. J. Davies , Org. Biomol. Chem. 2010, 8, 305. 20066263 10.1039/b915870g PMC2822703

[19] G. Caron , S. G. Withers , Biochem. Biophys. Res. Commun. 1989, 163, 495. 2673241 10.1016/0006-291x(89)92164-5

[20] L. I. Willems , T. J. M. Beenakker , B. Murray , B. Gagestein , H. van den Elst , E. R. van Rijssel , J. D. C. Codée , W. W. Kallemeijn , J. M. F. G. Aerts , G. A. van der Marel , H. S. Overkleeft , Eur. J. Org. Chem. 2014, 2014, 6044.

[21] L. I. Willems , J. Jiang , K. Li , M. D. Witte , W. W. Kallemeijn , T. J. N. Beenakker , S. P. Schröder , J. M. F. G. Aerts , G. A. van der Marel , J. D. C. Codée , H. S. Overkleeft , Chem. Eur. J. 2014, 20, 10864. 25100671 10.1002/chem.201404014

[22] B. P. Rempel , S. G. Withers , Glycobiology 2008, 18, 570. 18499865 10.1093/glycob/cwn041

[23] A. Aljoundi , I. Bjij , A. E. Rashedy , M. E. S. Soliman , Protein J. 2020, 39, 97. 32072438 10.1007/s10930-020-09884-2

[24] A. Tuley , W. Fast , Biochemistry 2018, 57, 3326. 29689165 10.1021/acs.biochem.8b00315 PMC6016374

[25] A. Alcaide , A. Trapero , Y. Pérez , A. Llebaria , Org. Biomol. Chem. 2015, 13, 5690. 25895752 10.1039/c5ob00532a

[26] T. P. Ofman , G. A. van der Marel , J. D. C. Codée , H. S. Overkleeft , Eur. J Org. Chem. 2023, 26, e202300186.

[27] P. V. Murphy , H. Bradley , M. Tosin , N. Pitt , G. M. Fitzpatrick , W. K. Glass , J. Org. Chem. 2003, 68, 5692. 12839465 10.1021/jo034336d

[28] P. Scheiner , J. Am. Chem. Soc. 1968, 90, 988.

[29] D. De Loera , M. A. Garcia‐Garibay , Org. Lett. 2012, 14, 3874. 22794188 10.1021/ol301582n

[30] S. Saryazdi , S. Parkin , R. B. Grossman , Org. Lett. 2023, 25, 331. 36626894 10.1021/acs.orglett.2c03908

[31] S. Xie , S. A. Lopez , O. Ramström , M. Yan , K. N. Houk , J. Am. Chem. Soc. 2015, 137, 2958. 25553488 10.1021/ja511457g PMC4351169

[32] R. Huisgen , L. Möbius , G. Müller , H. Stangl , G. Szeimies , J. M. Vernon , Chem. Ber. 1965, 98, 3992.

[33] D. A. Evans , M. T. Bilodeau , M. M. Faul , J. Am. Chem. Soc. 1994, 116, 2742.

[34] S. A. Lopez , K. N. Houk , J. Org. Chem. 2013, 78, 1778. 22764840 10.1021/jo301267b

[35] C. De Micheli , R. Gandolfi , R. Oberti , J. Org. Chem. 1980, 45, 1209.

[36] Gaussian 16, Revision C.02 , M. J. Frisch , G. W. Trucks , H. B. Schlegel , G. E. Scuseria , M. A. Robb , J. R. Cheeseman , G. Scalmani , V. Barone , G. A. Petersson , H. Nakatsuji , X. Li , M. Caricato , A. V. Marenich , J. Bloino , B. G. Janesko , R. Gomperts , B. Mennucci , H. P. Hratchian , J. V. Ortiz , A. F. Izmaylov , J. L. Sonnenberg , D. Williams‐Young , F. Ding , F. Lipparini , F. Egidi , J. Goings , B. Peng , A. Petrone , T. Henderson , D. Ranasinghe et al., Gaussian, Inc., Wallingford CT 2019, https://gaussian.com/citation/.

[37] M. Bols , R. G. Hazell , I. B. Thomsen , Chem. Eur. J. 1997, 3, 940.

[38] R. Eisenthal , A. Cornish‐Bowden , Biochem. J. 1974, 139, 715. 4854723 10.1042/bj1390715 PMC1166335

[39] A. Pesaresi , J. Enzyme Inhib. Med. Chem. 2023, 38, 2245168. 37577806 10.1080/14756366.2023.2245168 PMC10683834

[40] D. Voet , J. G. Voet , Biochemistry, Wiley, New York 1995.

[41] R. J. Desnick , Y. A. Ioannou , C. M. Eng , in The Metabolic and Molecular Bases of Inherited Disease (Eds: C. R. Scriver , A. L. Beaudet , W. S. Sly , D. Valle ), McGraw‐Hill, New York 2001, pp. 3733–3774.

[42] A. I. Guce , N. E. Clark , E. N. Salgado , D. R. Ivanen , A. A. Kulminskaya , H. Brumer , S. C. Garman , J. Biol. Chem. 2010, 285, 3625. 19940122 10.1074/jbc.M109.060145 PMC2823503

[43] L. Capaldo , Z. Wen , T. Noël , Chem. Sci. 2023, 14, 4230. 37123197 10.1039/d3sc00992k PMC10132167

[44] J. García‐Lacuna , G. Domínguez , J. Pérez‐Castells , ChemSusChem 2020, 13, 5138. 32662578 10.1002/cssc.202001372

[45] J. J. Bennett , P. V. Murphy , Carbohydr. Res. 2023, 529, 108845. 37210941 10.1016/j.carres.2023.108845

[46] F. Tropper , F. Anderson , S. Braun , R. Roy , Synthesis 1992, 7, 618.

[47] D. P. Thibodeaux , G. P. Johnson , E. D. Stevens , A. D. French , Carbohydr. Res. 2002, 337, 2301. 12433494 10.1016/s0008-6215(02)00266-5

[48] A. D. Becke , J. Chem. Phys. 1993, 98, 5648.

[49] R. Ditchfield , W. J. Hehre , J. A. Pople , J. Chem. Phys. 1971, 54, 724.

[50] A. V. Marenich , C. J. Cramer , D. G. Truhlar , J. Phys. Chem. B 2009, 113, 6378. 19366259 10.1021/jp810292n

[51] K. Wolinski , J. F. Hinton , P. Pulay , J. Am. Chem. Soc. 1990, 112, 8251.

