# Supplementary material for: Synthesis of an N‐Galactosyl Norbornane Aziridine and its Potent Mixed Inhibition of Aspergillus oryzae β‐Galactosidase
Source: Chembiochem. 2025 Oct 27;26(23):e202500623. doi: 10.1002/cbic.202500623 (PMC12666248; doi:10.1002/cbic.202500623)
Supplement: Supplementary file 1 — Supplementary Material [file CBIC-26-e202500623-s001.pdf]

Synthesis of an N-Galactosyl Norbornene Aziridine (NGNA) and its Potent Mixed Inhibition of *Aspergillus oryzae* β-Galactosidase

Aaron McCormack,[a,c] Ronan Gavin[a], Mikael Bols*[c] and Paul V. Murphy*[a,b]

[a] *A. McCormack, R. Gavin, Prof P.V. Murphy*
School of Biological and Chemical Sciences
University of Galway
University Rd, Galway, Ireland
E-mail: paul.v.murphy@universityofgalway.ie

[b] *SSPC Research Ireland Centre for Pharmaceuticals, University of Galway, University Rd. Galway, Ireland*

[c] *Prof. M. Bols*
Department of Chemistry
University of Copenhagen
Copenhagen, Denmark

Contents

General Experimental S2

Experimental section for synthesis of compound **2** S3

β-D-Galactosidase Inhibition Data S4 to S10

α-D-Galactosidase Inhibition Data S11 to S13

Calculations of ^{13}C NMR shift data S14 to S21

General Experimental Conditions: NMR spectra were recorded with a 400 MHz, a 500 MHz, and a 600 MHz spectrometer. NMR spectra were processed and analyzed using MestReNova software (v14.0.0-23239, mestrelab.com, Barcelona, Spain). Chemical shifts are reported relative to internal Me_4Si (TMS) in $CDCl_3$ (δ 0.0) for ^1H and Me_4Si in $CDCl_3$ (δ 77.0) for ^{13}C. Signals from ^1H and ^{13}C-NMR spectra were assigned with the aid of COSY, HSQC and HMBC. Coupling constants are reported as observed in hertz. The IR spectra were obtained using a PerkinElmer 100 FTIR spectrometer. High resolution mass spectra were obtained in positive mode using an Agilent UHPLC QTOF MSMS instrument. Reaction monitoring with thin layer chromatography (TLC) was performed on aluminium sheets precoated with silica gel 60 (HF_{245}, E. Merck, Merck, KGaA, Darmstadt, Germany), with spots visualized by UV and charring with H_2SO_4:MeOH (1:20). Column chromatography was carried out with silica gel 60 purchased from Sigma-Aldrich. All reagents used were obtained from commercial suppliers without further purification. CH_2Cl_2 and MeCN reaction solvents were used as obtained from a PureSolv™ Solvent Purification System. Cyclohexane, ethyl acetate (EtOAc), methanol and toluene were used as obtained from suppliers (Fisher, analytical reagent grade). Reactions were generally carried out under an atmosphere of N_2.

(2R,3S,4S,5R,6R)-2-(acetoxymethyl)-6-azidotetrahydro-2H-pyran-3,4,5-triyl triacetate or 2,3,4,6-tetra-O-acetyl-b-D-galactopyranosyl azide[27,48](2).

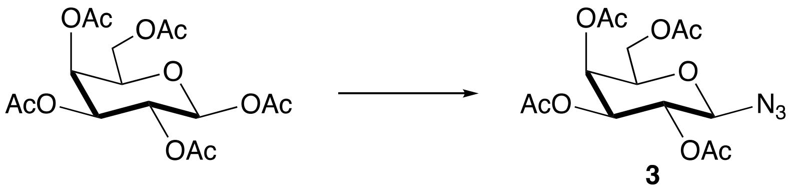

3

Compound **2** was prepared according to a reported procedure.[27] Thus, **β**-D-galactopyranose pentaacetate (15.00 g, 38.43 mmol) was suspended in dry dichloromethane (40 mL). Azidotrimethylsilane (5.05 mL, 38.4 mmol) and $SnCl_4$ (2.25 mL, 19.2 mmol) were added, and the reaction mixture was stirred at room temp. TLC analysis indicated that the reaction reached completion after 1 h. Saturated sodium bicarbonate solution (50 mL) was added, and the reaction mixture was allowed to stir for an additional 30 min. The mixture was washed with more saturated sodium bicarbonate solution (2 x 50 mL), water (3 x 50 mL) and dried over anhydrous sodium sulfate. The organic layer was concentrated in vacuo to afford the title compound as a white solid (12.1 g, 84%). ^{1}H-NMR data was in good agreement with literature values.[48]

NMR data for compound 2: ^{1}H-NMR (500 MHz, $CDCl_3$) *δ:* 5.42 (dd, 1H, $^3J_{4,5}$ 1.2 Hz, $^3J_{4,3}$ 3.4 Hz, H-4), 5.16 (dd, 1H, $^3J_{2,1}$ 8.8 Hz, $^3J_{2,3}$ 10.3 Hz, H-2), 5.04 (dd, 1H, $^3J_{3,4}$ 3.4 Hz, $^3J_{3,2}$ 10.3 Hz, H-3), 4.60 (d, 1H, $^3J_{1,2}$ 8.8 Hz, H-1), 4.17 (overlapping signals, 1H, H-6b), 4.17 (overlapping signals, 1H, H-6a), 4.01 (td, 1H, $^3J_{5,4}$ 1.2 Hz, $^3J_{5,6b}$ = $^3J_{5,6a}$ 6.5 Hz, H-5), 2.17 (s, 3H, OAc), 2.09 (s, 3H, OAc), 2.06 (s, 3H, OAc), 1.99 (s, 3H, OAc); ^{13}C-NMR (126 MHz, $CDCl_3$) *δ*: 170.53 (**C**=O, OAc), 170.26 (**C**=O, OAc), 170.15 (**C**=O, OAc), 169.52 (**C**=O, OAc), 88.46 (C-1), 73.03 (C-5), 70.88 (C-3), 68.21 (C-2), 66.99 (C-4), 61.37 (C-6), 20.82 ($\underline{C}H_3$, OAc), 20.81 ($\underline{C}H_3$, OAc), 20.76 ($\underline{C}H_3$, OAc), 20.67 ($\underline{C}H_3$, OAc);

β-Galactosidase Inhibition Assay Data – No Inhibitor

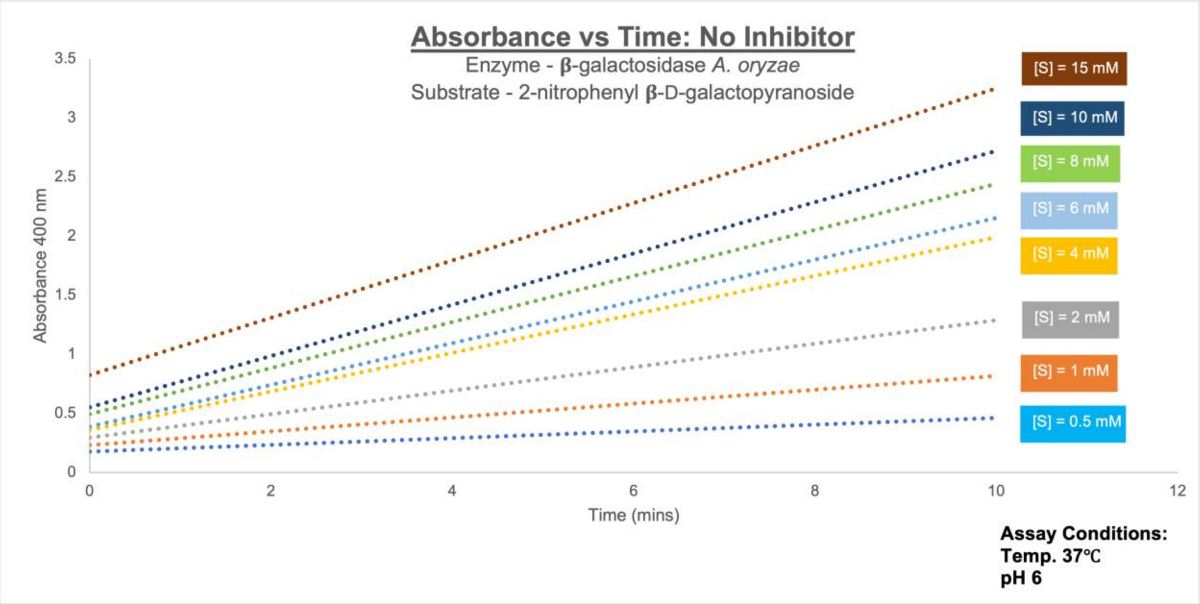

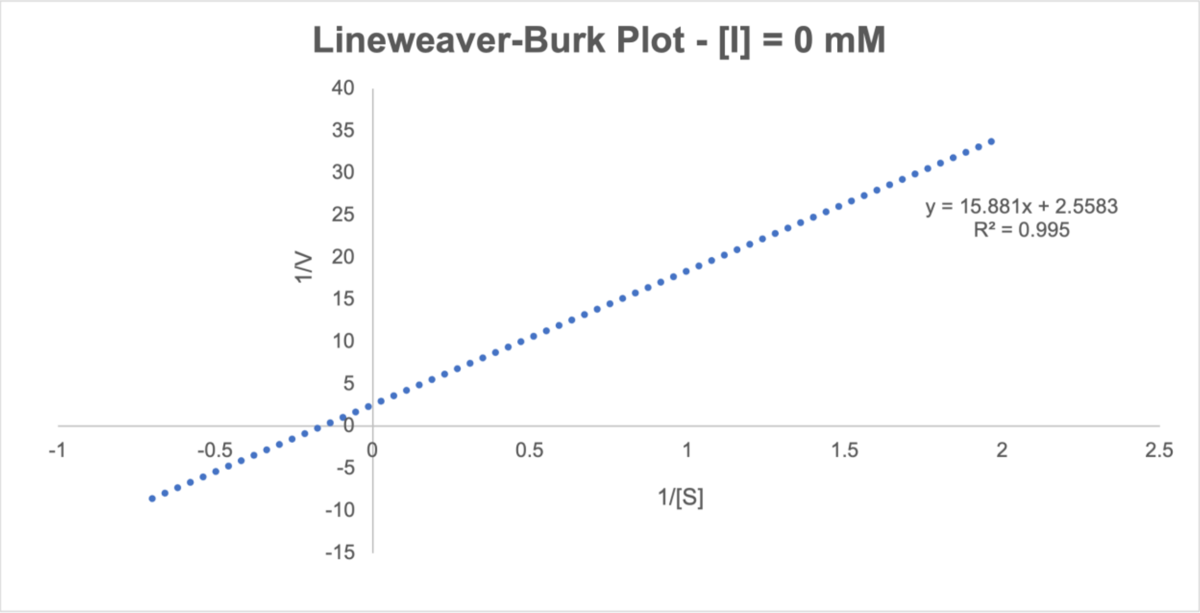

β-D-Galactosidase Inhibition Assay Data - Compound 6

When [I] = 2 mM

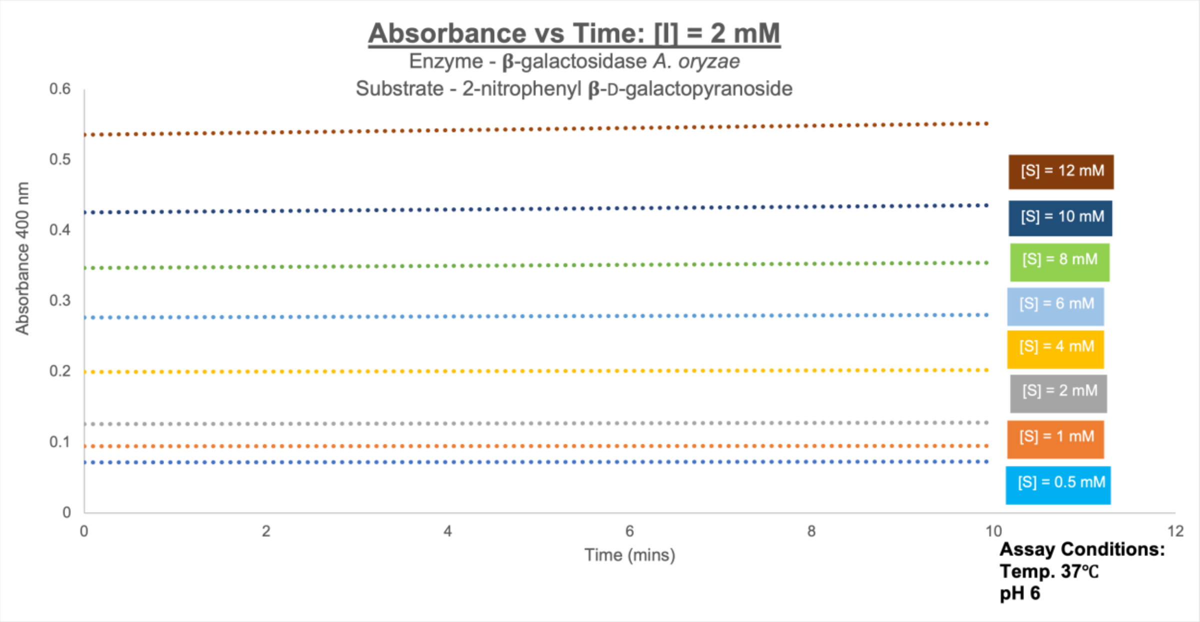

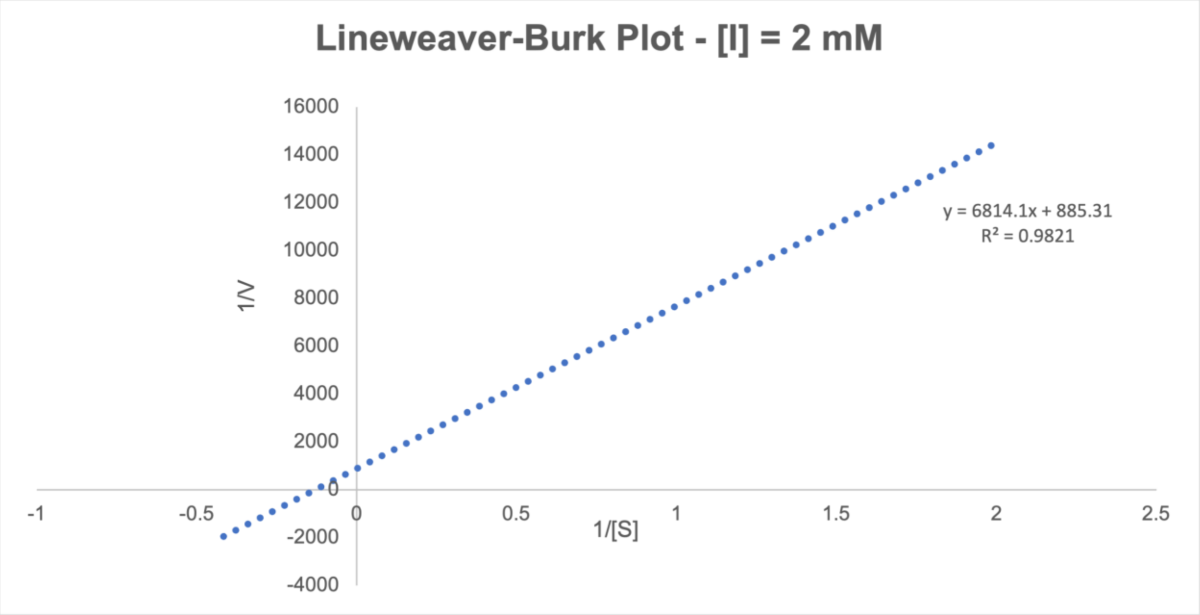

When [I] = 1 mM

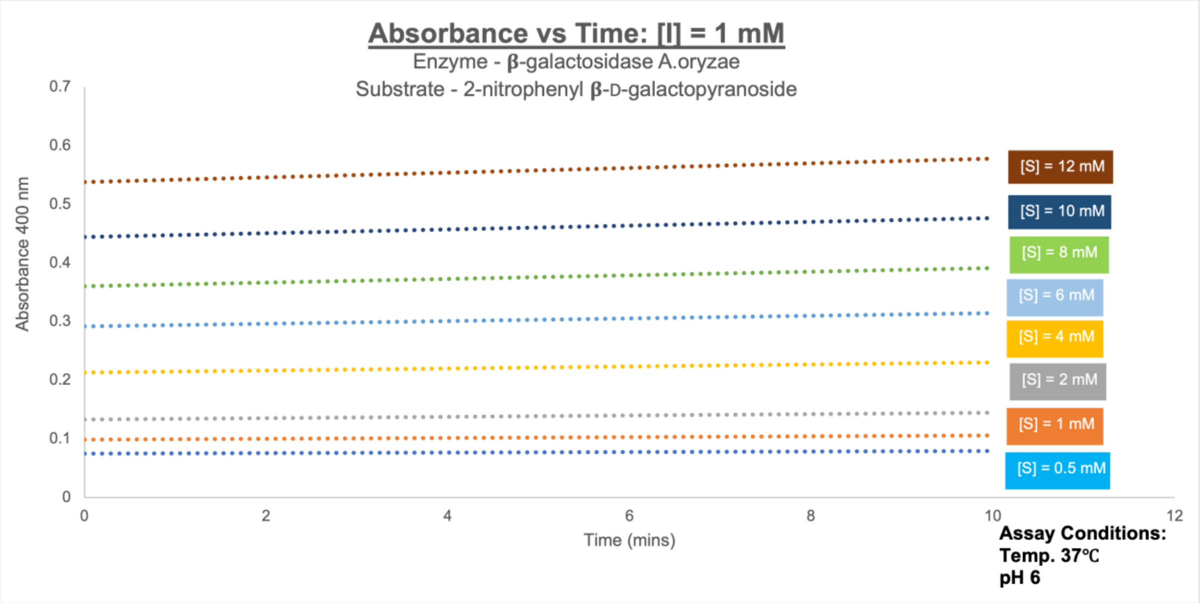

Absorbance vs Time: [I] = 1 mM
Enzyme - β-galactosidase A.oryzae
Substrate - 2-nitrophenyl β-D-galactopyranoside
Absorbance 400 nm
0.7
0.6
0.5
0.4
0.3
0.2
0.1
0
0
2
4
6
8
10
12
Time (mins)
[S] = 12 mM
[S] = 10 mM
[S] = 8 mM
[S] = 6 mM
[S] = 4 mM
[S] = 2 mM
[S] = 1 mM
[S] = 0.5 mM
Assay Conditions:
Temp. 37℃
pH 6

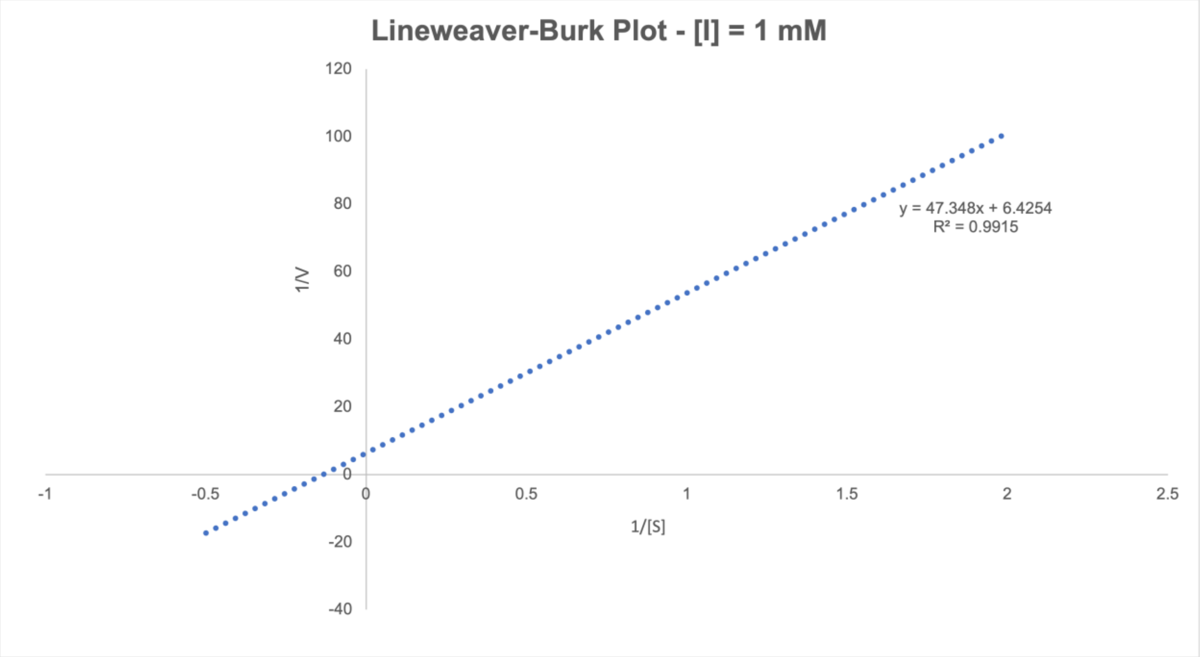

Lineweaver-Burk Plot - [I] = 1 mM
1/V
120
100
80
60
40
20
0
-20
-40
-1
-0.5
0
0.5
1
1.5
2
2.5
1/[S]
y = 47.348x + 6.4254
R² = 0.9915

When [I] = 0.1 mM

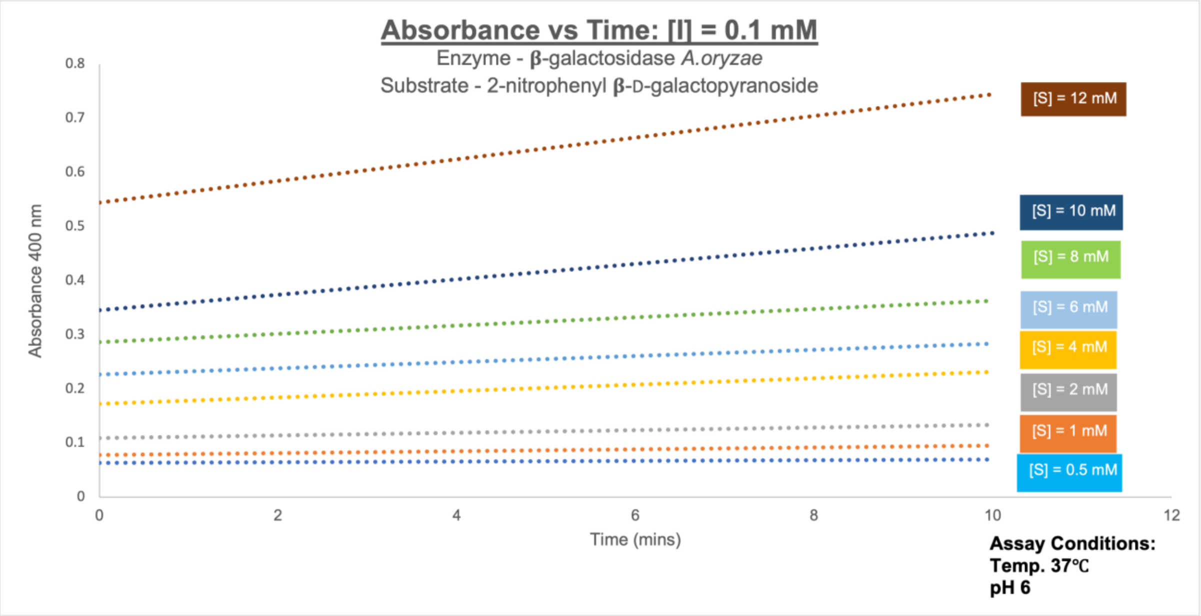
Absorbance vs Time: [I] = 0.1 mM
Enzyme - β-galactosidase A.oryzae
Substrate - 2-nitrophenyl β-D-galactopyranoside
Absorbance 400 nm
Time (mins)
[S] = 12 mM
[S] = 10 mM
[S] = 8 mM
[S] = 6 mM
[S] = 4 mM
[S] = 2 mM
[S] = 1 mM
[S] = 0.5 mM
Assay Conditions:
Temp. 37℃
pH 6

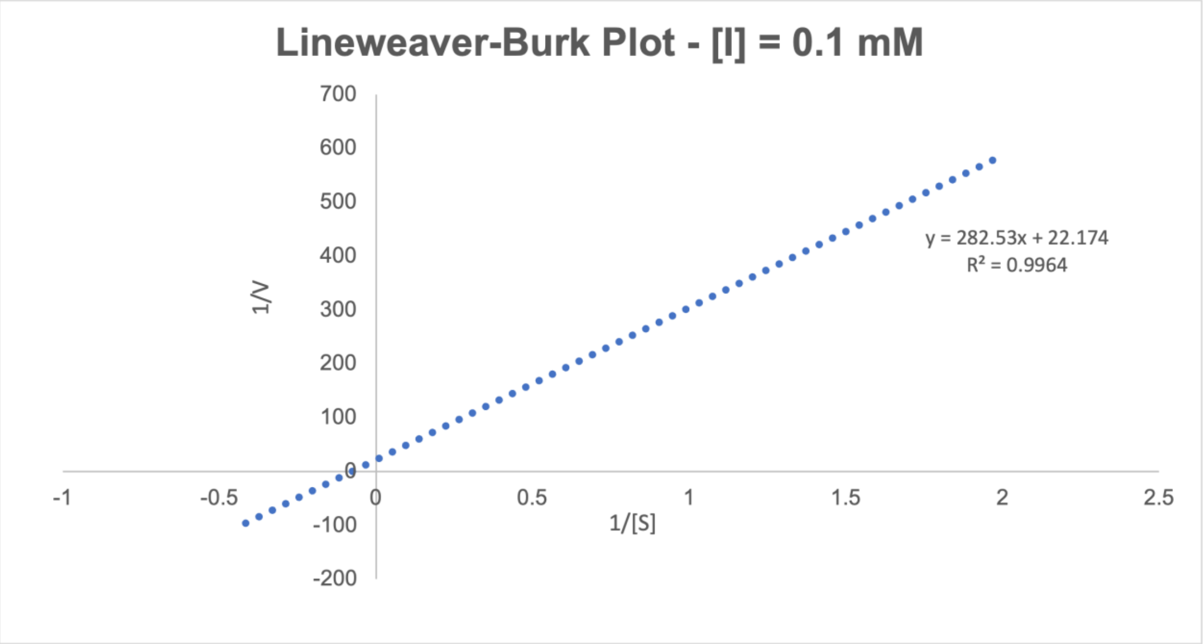
Lineweaver-Burk Plot - [I] = 0.1 mM
1/V
1/[S]
y = 282.53x + 22.174
R² = 0.9964

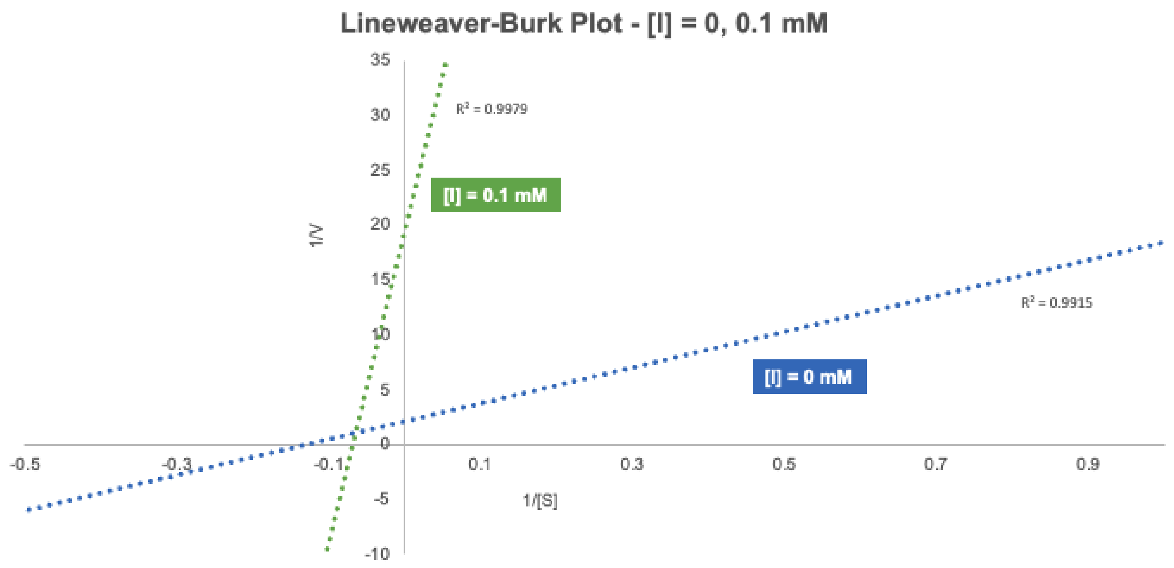

Lineweaver-Burk Plot - [I] = 0, 0.1 mM
1/V
1/[S]
R² = 0.9979
R² = 0.9915
[I] = 0.1 mM
[I] = 0 mM
35
30
25
20
15
10
5
0
-5
-10
-0.5
-0.3
-0.1
0.1
0.3
0.5
0.7
0.9

When [I] = 0.01 mM

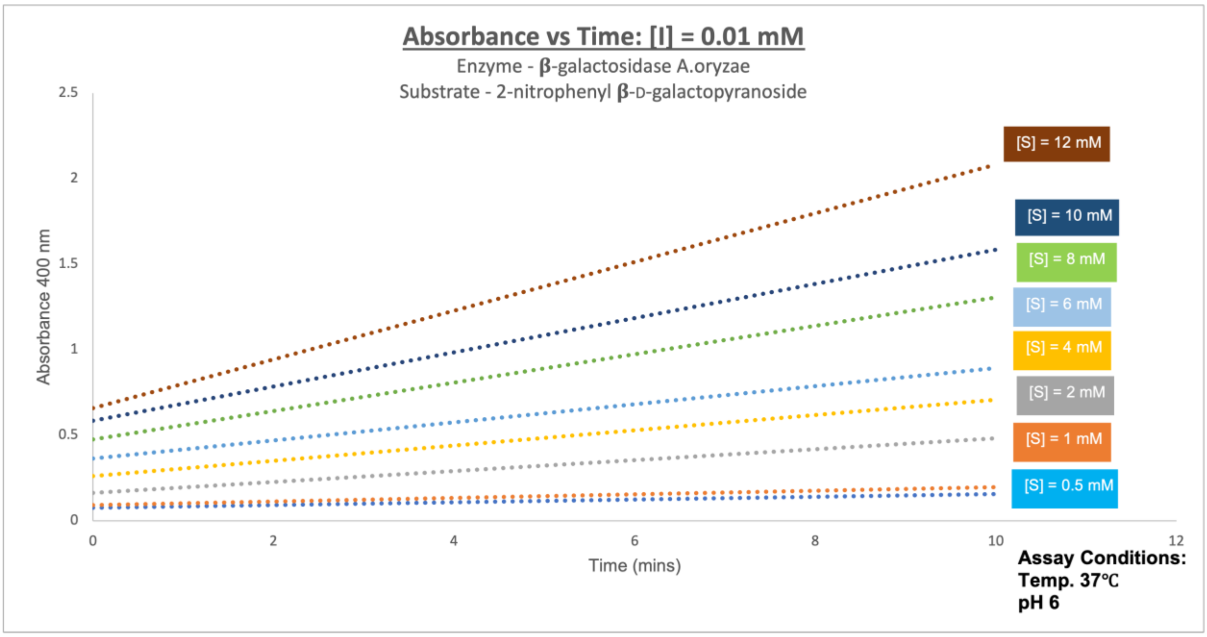
Absorbance vs Time: [I] = 0.01 mM
Enzyme - β-galactosidase A.oryzae
Substrate - 2-nitrophenyl β-D-galactopyranoside
Absorbance 400 nm
Time (mins)
[S] = 12 mM
[S] = 10 mM
[S] = 8 mM
[S] = 6 mM
[S] = 4 mM
[S] = 2 mM
[S] = 1 mM
[S] = 0.5 mM
Assay Conditions:
Temp. 37℃
pH 6

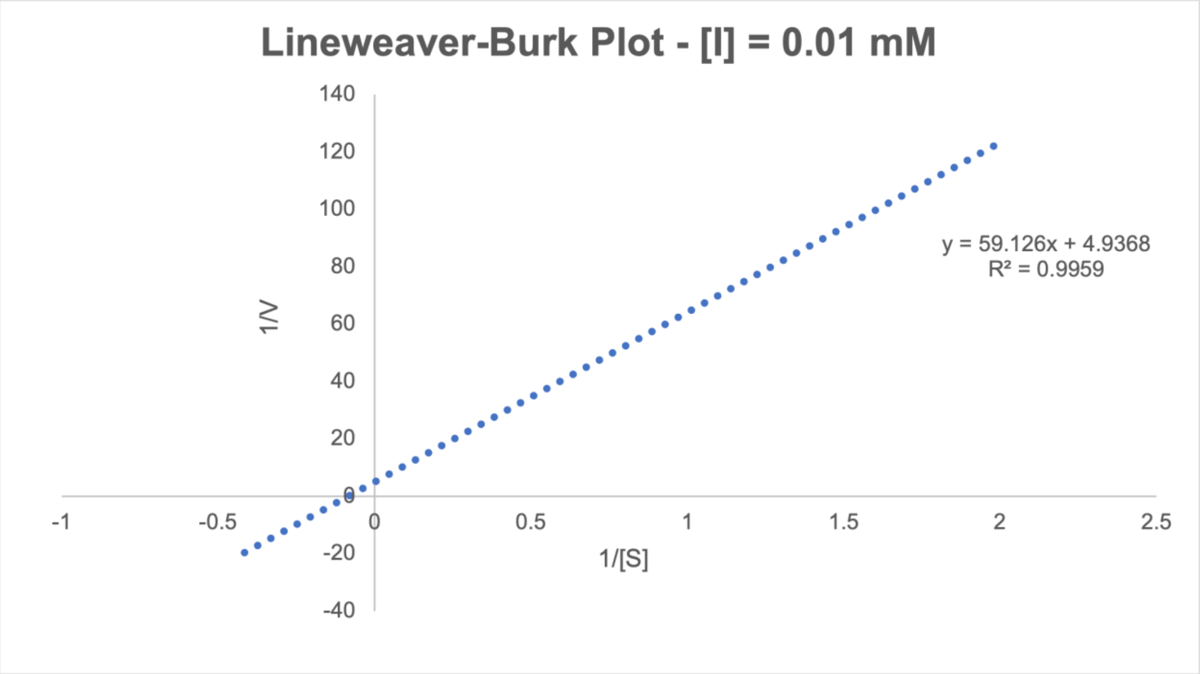
Lineweaver-Burk Plot - [I] = 0.01 mM
1/V
1/[S]
y = 59.126x + 4.9368
R² = 0.9959

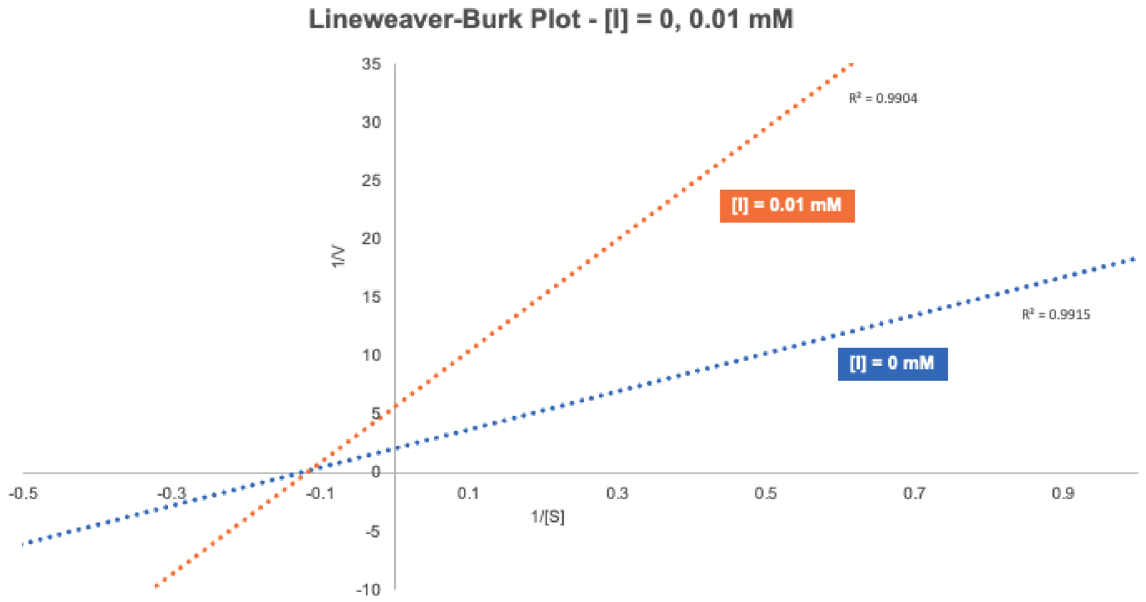
Lineweaver-Burk Plot - [I] = 0, 0.01 mM
R² = 0.9904
[I] = 0.01 mM
R² = 0.9915
[I] = 0 mM
1/V
1/[S]
35
30
25
20
15
10
5
0
-5
-10
-0.5
-0.3
-0.1
0.1
0.3
0.5
0.7
0.9

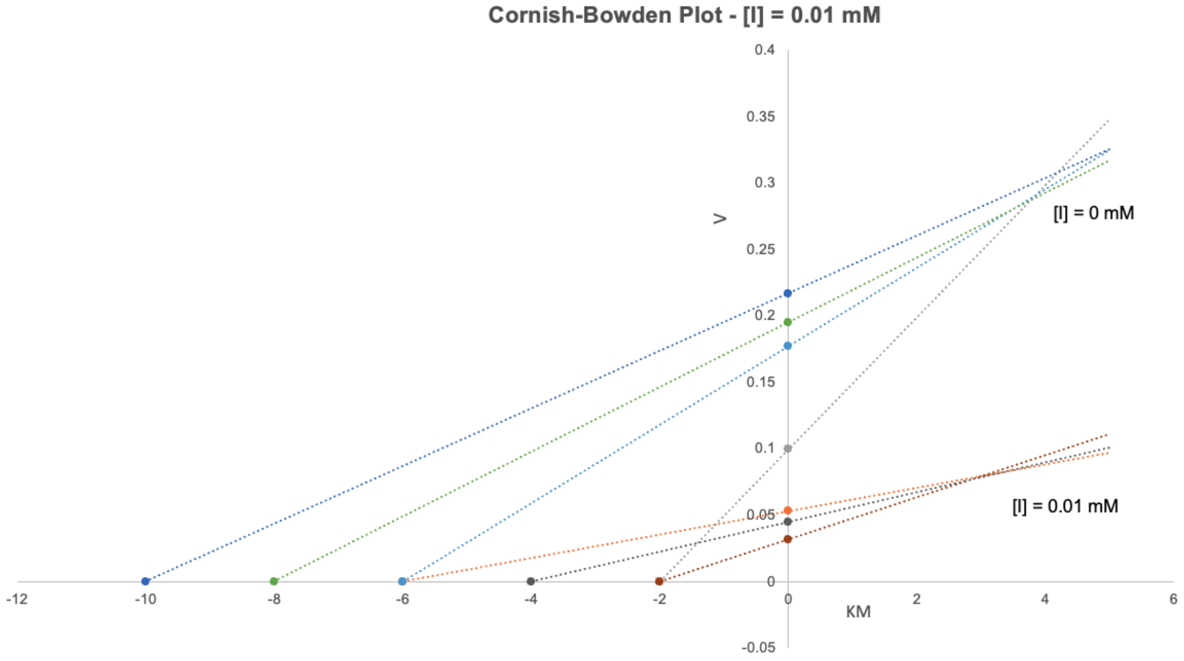
Cornish-Bowden Plot - [I] = 0.01 mM
V
KM
[I] = 0 mM
[I] = 0.01 mM
0.4
0.35
0.3
0.25
0.2
0.15
0.1
0.05
0
-0.05
-12
-10
-8
-6
-4
-2
0
2
4
6

α-Galactosidase Inhibition Assay – No Inhibitor

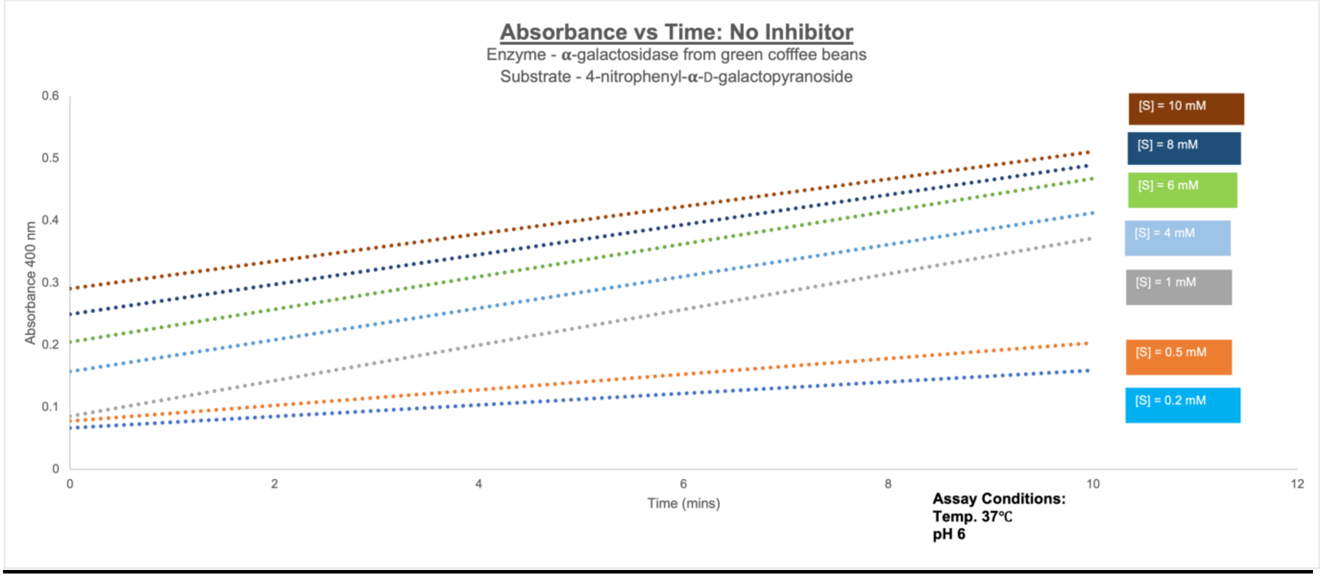

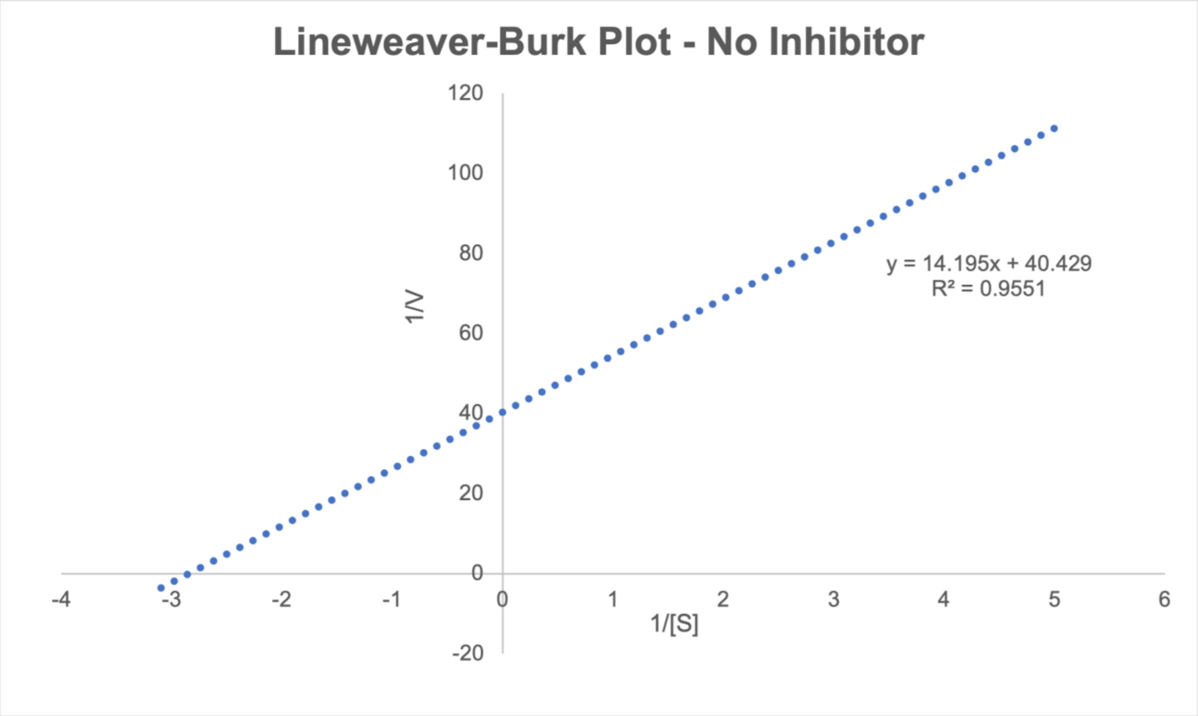

α-D-Galactosidase Inhibition Assay – Compound 6

When [I] = 1 mM

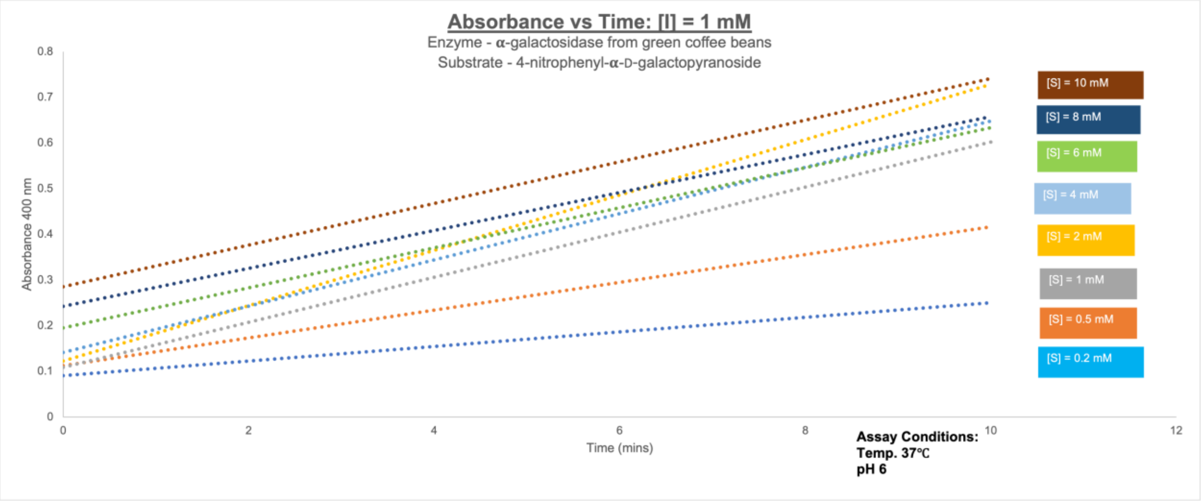

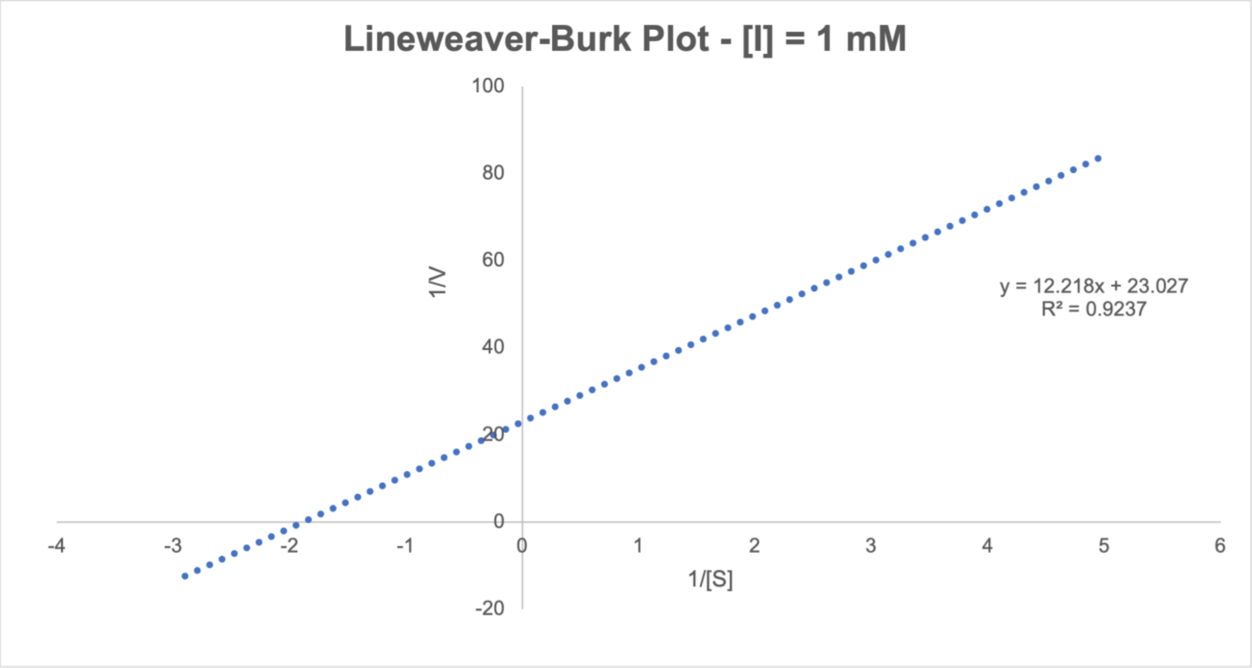

When [I] = 0.1 mM

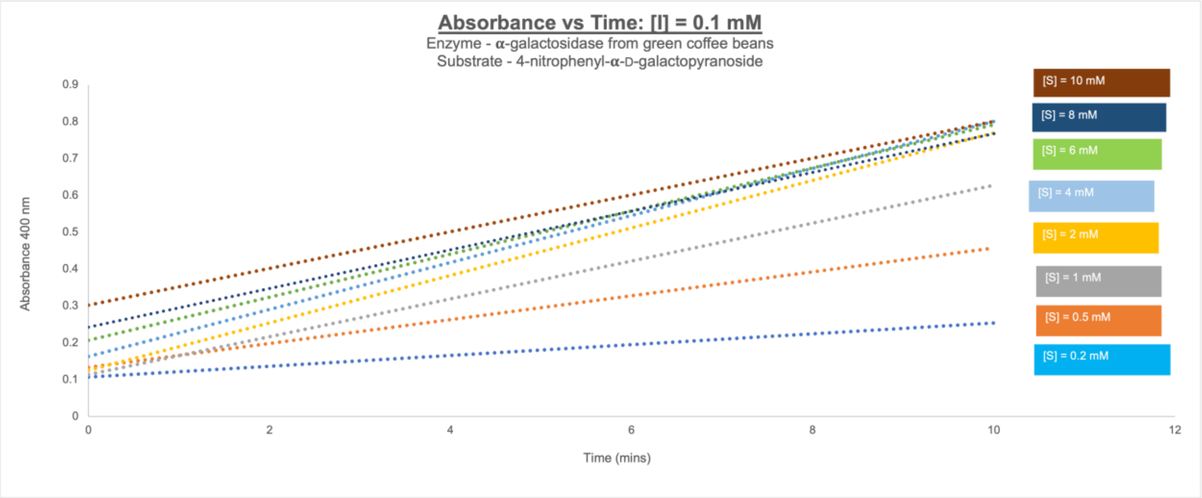
Absorbance vs Time: [I] = 0.1 mM
Enzyme - α-galactosidase from green coffee beans
Substrate - 4-nitrophenyl-α-D-galactopyranoside
Absorbance 400 nm
Time (mins)
[S] = 10 mM
[S] = 8 mM
[S] = 6 mM
[S] = 4 mM
[S] = 2 mM
[S] = 1 mM
[S] = 0.5 mM
[S] = 0.2 mM

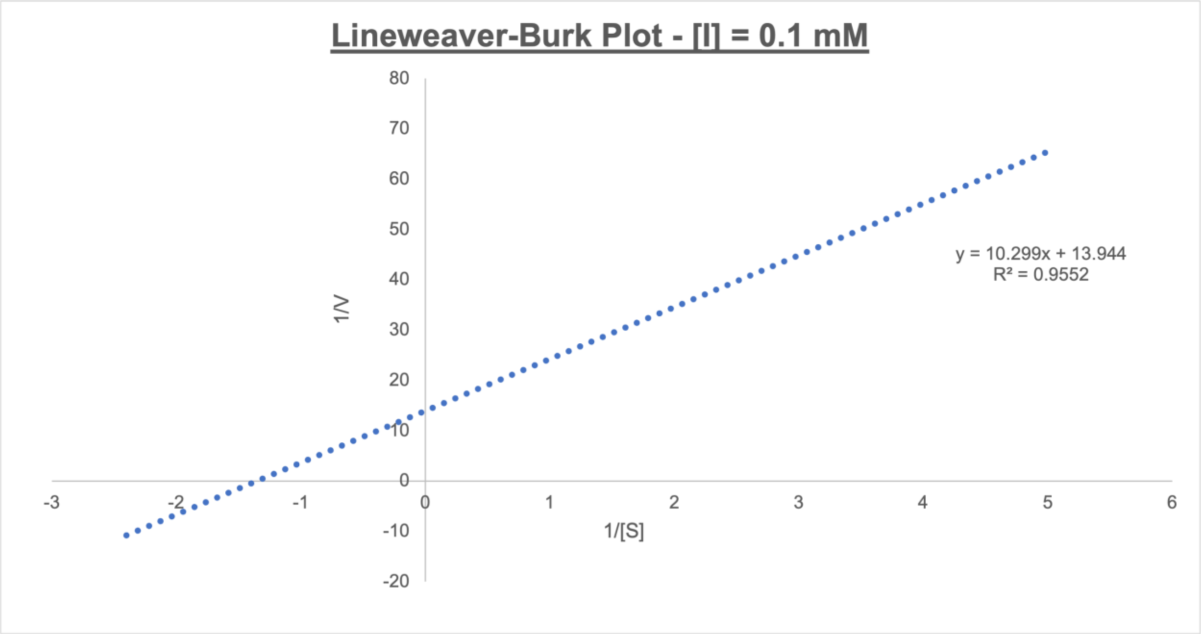
Lineweaver-Burk Plot - [I] = 0.1 mM
1/V
1/[S]
y = 10.299x + 13.944
R² = 0.9552

DFT calculation of ^{13}C chemical shifts

Geometries for **4-6** were first subjected to a conformational search using macromodel programme as implemented in Maestro (Schrodinger.com). The arrangement of OAc groups was adjusted based on published crystal structures of acetylated galactopyranosyl derivatives.[49] The ω torsion (defined by pyranose ring oxygen, C-5, C-6, O-6) was arranged in the gt torsional preference for the galactopyranoside. The shifts are calculated for one conformation only. It is usually recommended to calculate for all populated conformations. However, for the requirements for this manuscript and the rigidity of the norbornane residue it was deemed sufficient to predict for one conformer. Thus, geometries generated were optimized using Gaussian 16, Revision C.02.[36] using the B3LYP functional[50] and 6-311+G(2d,p) basis set[51] and a solvation model (solvation model based on density, SMD or smd,)[52] using chloroform as the solvent. NMR data was then calculated for the optimized conformer using the Gauge-Independent Atomic Orbital (GIAO) method[53] with spin-spin coupling, and the solvation model; the same functional and basis set was used again. The NMR results were analysed using Gaussview and predicted ^{13}C shifts, with reference to TMS (tetramethylsilane) calculated by GIAO using B3LYP/6-311+G(2d,p), also used for the optimization and chemical shift calculations. The reports generated by Gaussview are given below.

DFT calculated ^{13}C-NMR chemical shift data for compound 6 (exo isomer, atom labels shown)

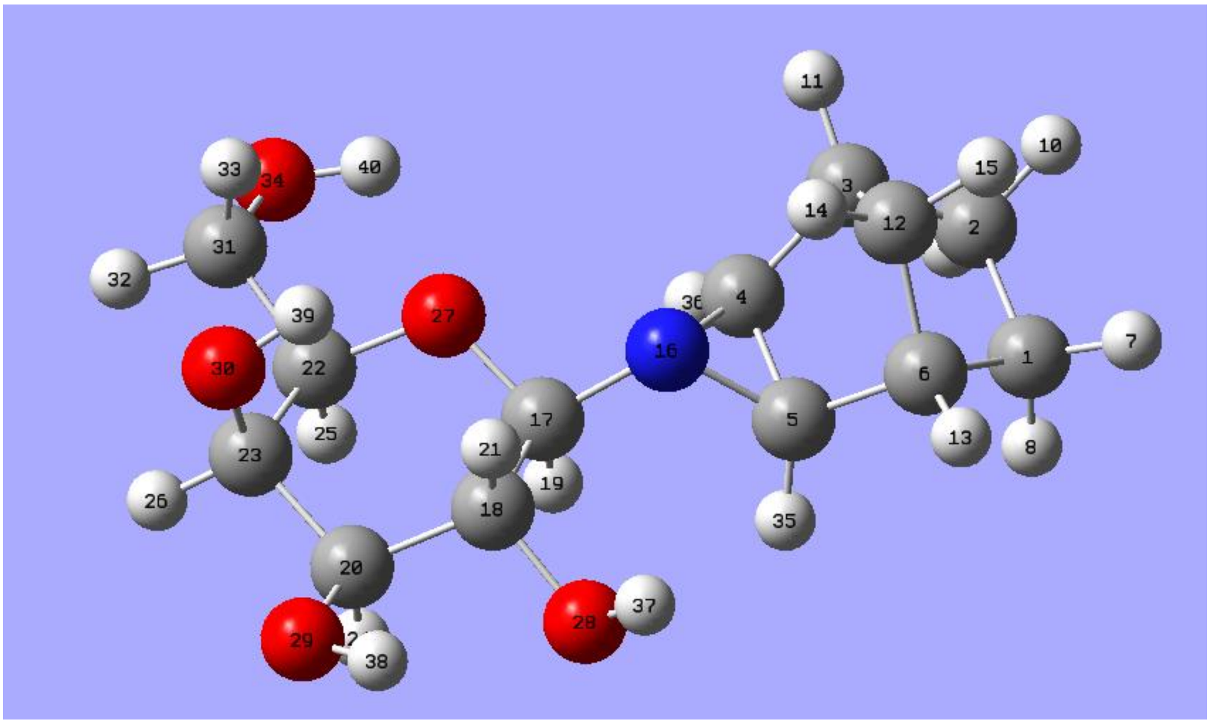

SCF GIAO Method (B3LYP/6-311+G(2d,p), scrf(smd,solvent=chloroform)

X-Axis: Calculated Shift (ppm)

Y-Axis: Degeneracy (no of carbons)

Reference: TMS calculated by B3LYP/6-311+G(2d,p) GIAO

Reference shielding: 182.466 ppm

NMR Degeneracy Tolerance: 0.05

#	Predicted shift	Y	Degeneracy	Assignment	Experimental	Diff.
C17	100.3789000000	1.0000000000	1.0000000000	C-1 anomeric	95.5	4.9
C22	82.5389000000	1.0000000000	1.0000000000	Gal C-5	78.3	4.2
C18	80.8571000000	1.0000000000	1.0000000000	Gal C-2	73.5	7.4
C20	78.9540000000	1.0000000000	1.0000000000	Gal C-3	75.5	3.5
C23	75.7543000000	1.0000000000	1.0000000000	Gal C-4	70.6	5.2
C31	70.5800000000	1.0000000000	1.0000000000	Gal C-6	62.7	7.9
C5	43.6667000000	1.0000000000	1.0000000000	Aziridine CH	39.2	4.5

C3	41.0356000000	1.0000000000	1.0000000000		37.3	3.7
C6	40.8998000000	1.0000000000	1.0000000000		37.1	3.8
C4	40.6705000000	1.0000000000	1.0000000000	Aziridine CH	37.4	3.3
C12	30.9666000000	1.0000000000	1.0000000000	Bridgehead C	29.3	1.7
C2	29.2851000000	1.0000000000	1.0000000000		27.7	1.6
C1	29.1512000000	1.0000000000	1.0000000000		27.5	1.7

Calculated ^{13}C-NMR spectrum and data for compound 6 (endo isomer, atom labels shown)

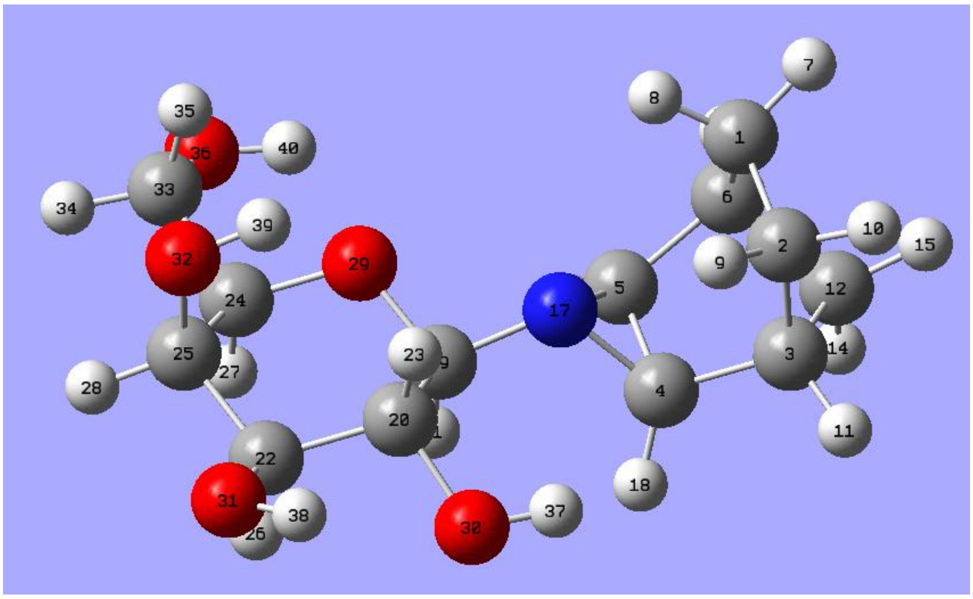

#	Predicted (ppm)	Degeneracy	Atoms	Assignment	Experimental	Difference
	99.8189000000	1.0000	19	Gal C-1	95.5	4.3
	82.6440000000	1.0000	24	Gal C-5	78.3	4.3
	81.0257000000	1.0000	20	Gal C-2	73.5	7.5
	79.7840000000	1.0000	22	Gal C-3	75.5	4.3
	75.5756000000	1.0000	25	Gal C-4	70.6	4.0
	70.5022000000	1.0000	33	Gal C-6	62.7	7.3
	53.7021000000	1.0000	12	Bridging C	29.3	23.5
	50.3013000000	1.0000	4	Aziridine CH	39.2	11.3
	47.8892000000	1.0000	5	Aziridine CH	37.4	10.4
	42.3254000000	2.0000	6,3		37.3,1	5, 4.8
	29.3381000000	1.0000	2		27.7	1.6
	29.0319000000	1.0000	1		27.5	1.5

Calculated NMR data for triazoline 1 diastereoisomer 4/5

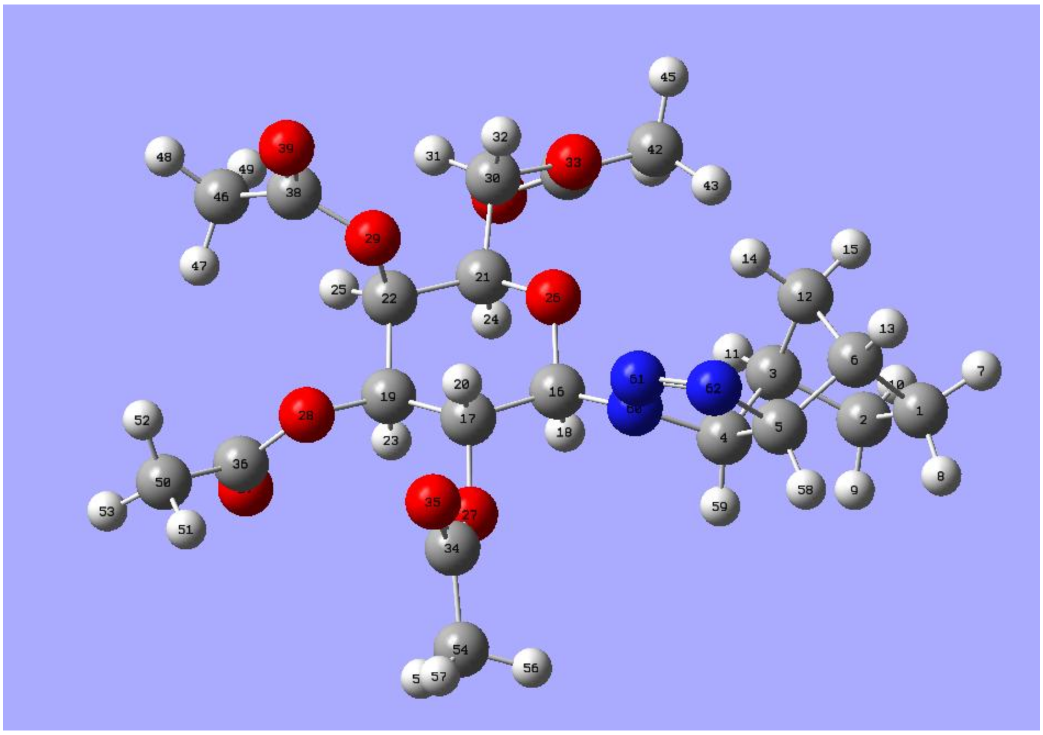

Summary of NMR spectra (SCF GIAO Method)

Values for element C only

Reference: TMS B3LYP/6-311+G(2d,p) GIAO [scrf(smd,solvent=chloroform)]

Reference shielding: 182.466 ppm

Degenerate peaks are condensed together (Degeneracy Tolerance 0.05)

#	Predicted (ppm)	Degeneracy	Atoms	Assignment	Experimental (4/5)	Diff
	181.0085000000	1.0000	40	C=O		
	180.0362000000	1.0000	36	C=O		
	178.2616000000	1.0000	38	C=O		
	177.7437000000	1.0000	34	C=O		
	91.1622000000	1.0000	5	Triazoline C	87.37/89.85	3.79/1.31
	89.3366000000	1.0000	16	Gal C-1	86.41/86.35	2.93/2.99
	79.7864000000	1.0000	22			
	78.6786000000	1.0000	21			
	76.7633000000	1.0000	19			
	70.6153000000	1.0000	17			
	67.8670000000	1.0000	30			

67.2666000000	1.0000	4	Triazoline C	59.14/57.21	8.13/10.06
48.0154000000	1.0000	3			
46.0382000000	1.0000	6			
33.8899000000	1.0000	12	Bridging C.	33.38/32.34	0.51/1.55
28.5488000000	1.0000	1			
28.0298000000	1.0000	2			
20.8321500000	2.0000	42,50			
20.6885000000	1.0000	54			
19.4200000000	1.0000	46			

Calculated NMR data for triazoline 2 diastereoisomer 4/5

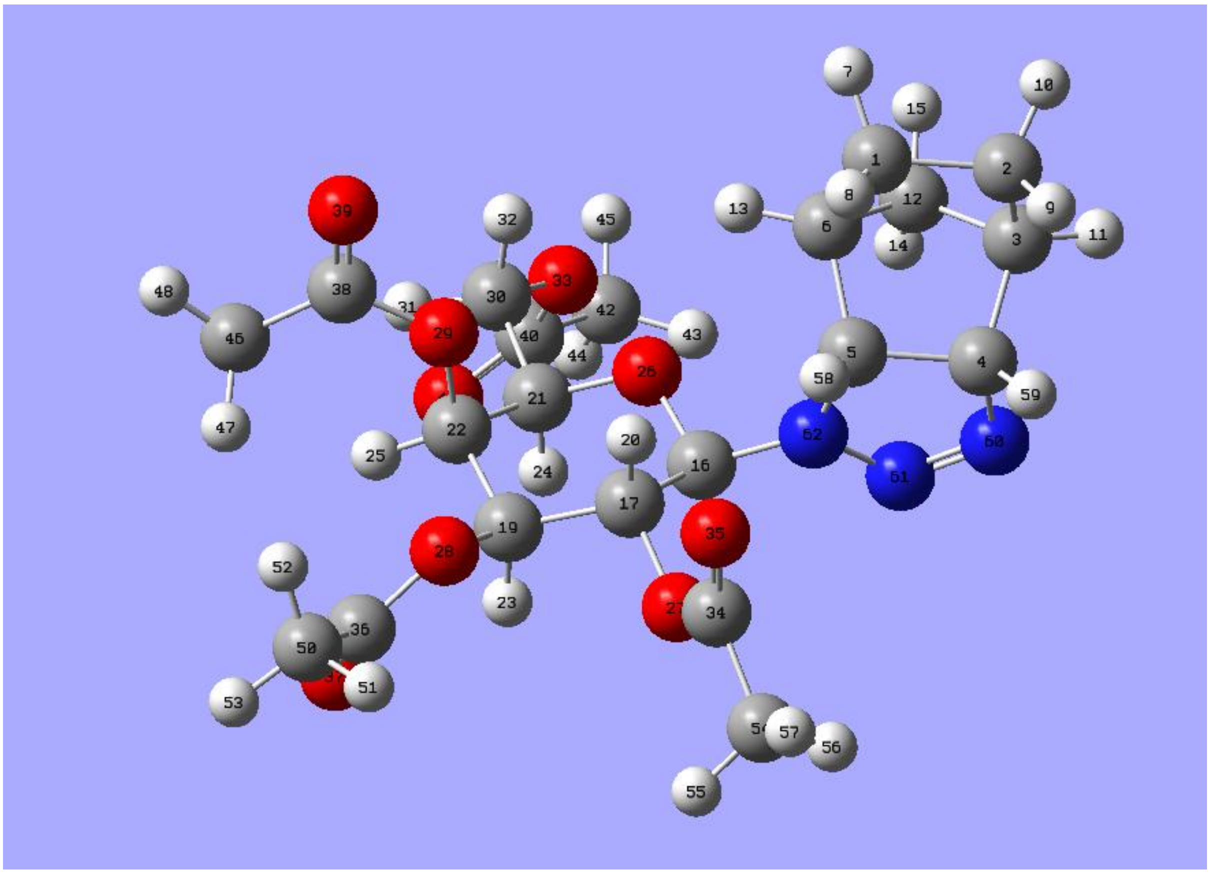

Summary of NMR spectra (SCF GIAO Method)

Values for element C only

Reference: TMS B3LYP/6-311+G(2d,p) GIAO

Reference shielding: 182.466 ppm

Degenerate peaks are condensed together (Degeneracy Tolerance 0.05)

#

# Shift (ppm)	Degeneracy	Atoms	Assignment	Experimental 4/5.	Difference
180.9147000000	1.0000	40	C=O		
180.2123000000	1.0000	36	C=O		
179.1056000000	1.0000	34	C=O		
178.6400000000	1.0000	38	C=O		
92.7283000000	1.0000	4	triazoline CH	87.37/89.85	5.36/2.88
91.5412000000	1.0000	16	Gal C1	86.41/86.35	5.13/5.19
79.3043000000	1.0000	22			
78.5506000000	1.0000	21			

77.2607000000	1.0000	19			
68.8025000000	1.0000	17			
67.3798000000	1.0000	30			
62.2618000000	1.0000	5	Triazoline CH	59.14/57.21	3.12/5.05
48.0872000000	1.0000	6			
46.4966000000	1.0000	3			
33.9580000000	1.0000	12	Bridging C	33.38/32.34	0.58/1.62
28.5307000000	1.0000	2			
27.8394000000	1.0000	1			
20.8078000000	1.0000	42			
20.7102000000	2.0000	54,50			
19.3564000000	1.0000	46			
